# Identification of novel H2A histone variants across diverse clades of algae

**DOI:** 10.1186/s13059-025-03656-w

**Published:** 2025-09-23

**Authors:** Ellyn Rousselot, Zofia Nehr, Jean-Marc Aury, France Denoeud, J. Mark Cock, Leïla Tirichine, Céline Duc

**Affiliations:** 1 Nantes Université, CNRS, US2B UMR 6286, 44000 Nantes, France; 2 Laboratory of Integrative Biology of Marine Models, Sorbonne Université, UPMC University of Paris 06, CNRS, UMR 8227, Station Biologique de Roscoff, CS 90074, Roscoff, 29688 France; 3 Génomique Métabolique, Genoscope, Institut François Jacob, CEA, CNRS, Université Evry, Université Paris-Saclay, Evry, 91057 France; 4 Institute for Marine and Antarctic Studies (IMAS), Ecology and Biodiversity Centre, University of Tasmania, TAS, Hobart, 7004 Australia

**Keywords:** H2A, Histone variants, Brown seaweeds, Algal evolution, Transcriptional regulation

## Abstract

**Background:**

Histones are among the most conserved proteins in eukaryotes. They not only ensure DNA compaction in the nucleus but also participate in epigenetic regulation of gene expression. These key epigenetic players are divided into replication-coupled histones, expressed during the S-phase, and replication-independent variants, expressed throughout the cell cycle. Compared with other core histones, H2A proteins exhibit a high level of variability but the characterization of algal H2A variants remains very limited.

**Results:**

In this study, we exploit genome and transcriptome data from 22 species to identify H2A variants in brown seaweeds. Combined analyses of phylogenetic data, synteny and protein motifs enable us to reveal the presence of new H2A variants as well as their evolutionary history. We identify three new H2A variants: H2A.N, H2A.O and H2A.E. In brown seaweeds, the H2A.E and H2A.O variants arose from the same monophyletic clade while the H2A.N variant emerged independently. Moreover, the H2A.E variant seems to have a shared ancestry with RC H2A while the H2A.O variant has an H2A.X-characteristic signature without being orthologous to this variant. Based on mass spectrometry, we identify distinct epigenetic marks on these H2A variants. Finally, the H2A.Z, H2A.N and H2A.O from brown seaweeds are ubiquitously expressed while expression of H2A.E has tissue-specific patterns, especially in reproductive tissues.

**Conclusions:**

We thus hypothesize that H2A.O and H2A.X might have convergent functions while H2A.E might fulfil some functions of replication-coupled H2As and/or compensate for the absence of repressive histone marks along with H2A.N.

**Supplementary Information:**

The online version contains supplementary material available at 10.1186/s13059-025-03656-w.

## Background

In eukaryotes, DNA is compacted into chromatin within the nucleus. The basic subunit of chromatin is the nucleosome, which is composed of ~147bp of DNA wrapped around a histone octamer [1]. The H3-H4 tetramer is associated with two H2A-H2B dimers to compose the histone core. Core histones (*i.e.* RC H2A, H2B, H3.1 and H4) have amino- and carboxy-terminal tails harboring various post-translational modifications that contribute to epigenetic information and a characteristic domain known as the histone-fold domain. This histone fold consists of three α-helices (α1, α2, and α3) connected by short loops L1 and L2 [1]. Histone proteins can differ in the number of variable amino acids to different extents. Histones are classified into two main categories: (i) replication-coupled (RC) variants or canonical histones, which are highly expressed during the S-phase and (ii) replication-independent (RI) variants, also called replacement histone variants, which are expressed throughout the cell cycle [2]. Histone H2A participates in nucleosome stability and DNA compaction and has specialized variants. Indeed, most eukaryotes have at least three histone variants, RC H2A plus RI H2A.X and H2A.Z variants. While the biological functions of RC H2A remain elusive, RI H2A variants are actively studied. The H2A.X variant is a marker of DNA damage events and is involved in a wide variety of cellular processes such as chromosome segregation or development [3], indicating a general role in genome stability. The H2A.Z variant is involved in various mechanisms such as transcription regulation, DNA repair, differentiation and is linked to several cancers associated with epigenetic disorders (reviewed in [4, 5]).

All H2A variants have an acidic patch composed of acidic residues located in their C-terminal tails and in the α2 helix. These H2A acidic residues, together with those of H2B, interact with the H4 N-terminal tail [1]. In addition, the docking domain, which includes the α3 and αC helices, enables interaction with the H4 C-terminal tail [1]. H2A.X is characterized by a SQ[D/E] [F/Y] motif at the very end of its C-terminal tail. The serine in this motif is phosphorylated in response to double-strand DNA breaks in animals and yeast [6] as well as in plants [7]. H2A.Z harbors a LEYLTAEVLELAGNA signature in the α2 helix [8] and an L1 loop with a single amino acid insertion, an acidic patch with an extra acidic residue and a docking domain that is one amino acid shorter. Additional RI H2A variants have been identified in some eukaryotes. For example, vertebrates have a macroH2A protein harboring a macro-domain in its C-terminal tail and mammals have several additional RI H2A variants, namely H2A.B, H2A.P and H2A.L. Seed-bearing plants have a characteristic H2A variant, H2A.W, which possesses a stretch of lysine and glycine residues in its N-terminal tail, a RY[A/S][Q/K] sequence in its L1 loop [9] and a KSPKK C-terminal motif: this variant has a critical role in heterochromatin formation [10, 11]. In addition, land plants other than Angiosperms have another specific variant, H2A.M. This variant, which is present in the liverwort *Marchantia polymorpha* and the moss *P. patens*, is characterized by a RYA[Q/K] sequence in its L1 loop [9].

Few studies have identified histones in algae [12–16] consequently leaving an important knowledge gap regarding algal histones. The investigation of histone variants in algae is relatively limited so far due to the considerable diversity within this group, which includes both unicellular and multicellular organisms spanning diverse independent phylogenetic clades, and due to the limited availability of genomic and transcriptomic data. There are three main clades of algae (Rhodophyta, Chlorophyta and photosynthetic Stramenopiles). Rhodophyta and Chlorophyta belong to the kingdom Archaeplastida, a major lineage of photosynthetic organisms that also includes land plants while the brown algae evolved independently within the distantly related Stramenopile lineage. Until recently, a limited number of genomes has been available for algal species but the recent release of the Phaeoexplorer dataset has provided high quality reference genomes for multiple brown seaweeds [17]. Using these data, we carried out detailed phylogenetic analyses of histone H2A variants in brown seaweeds and identified three novel H2A variants, which we named H2A.N, H2A.O, H2A.E. These newly identified H2A variants are either specific to brown seaweeds (H2A.N) or also found in the green and red algal lineages (H2A.E and H2A.O). While the brown seaweed-specific variant, H2A.N, constitutes a monophyletic and independent clade, the H2A.E and H2A.O variants emerged from a shared ancestor in brown seaweeds. The three novel H2A variants have several sequence divergences from other H2A variants: the H2A.N variant has an elongated N-terminal tail and shares some features with RC H2As regarding its C-terminal tail and the H2A.O has the H2A.X signature in its C-terminal tail. H2A.E genes are localized in histone gene clusters. Whereas H2A.Z, H2A.N and H2A.O variants are expressed at most life cycle stages, the H2A.E variants appeared to be preferentially expressed in reproductive tissues. Altogether, our results suggest lineage-specific properties of H2A variants with, for example, H2A.E and/or H2A.N potentially compensating for the absence of repressive epigenetic marks, as well as putative evolutionary convergence between H2A.X and H2A.O.

## Results

### In-depth analysis of brown algal genomes to identify H2A variants

**Fig. 1:**
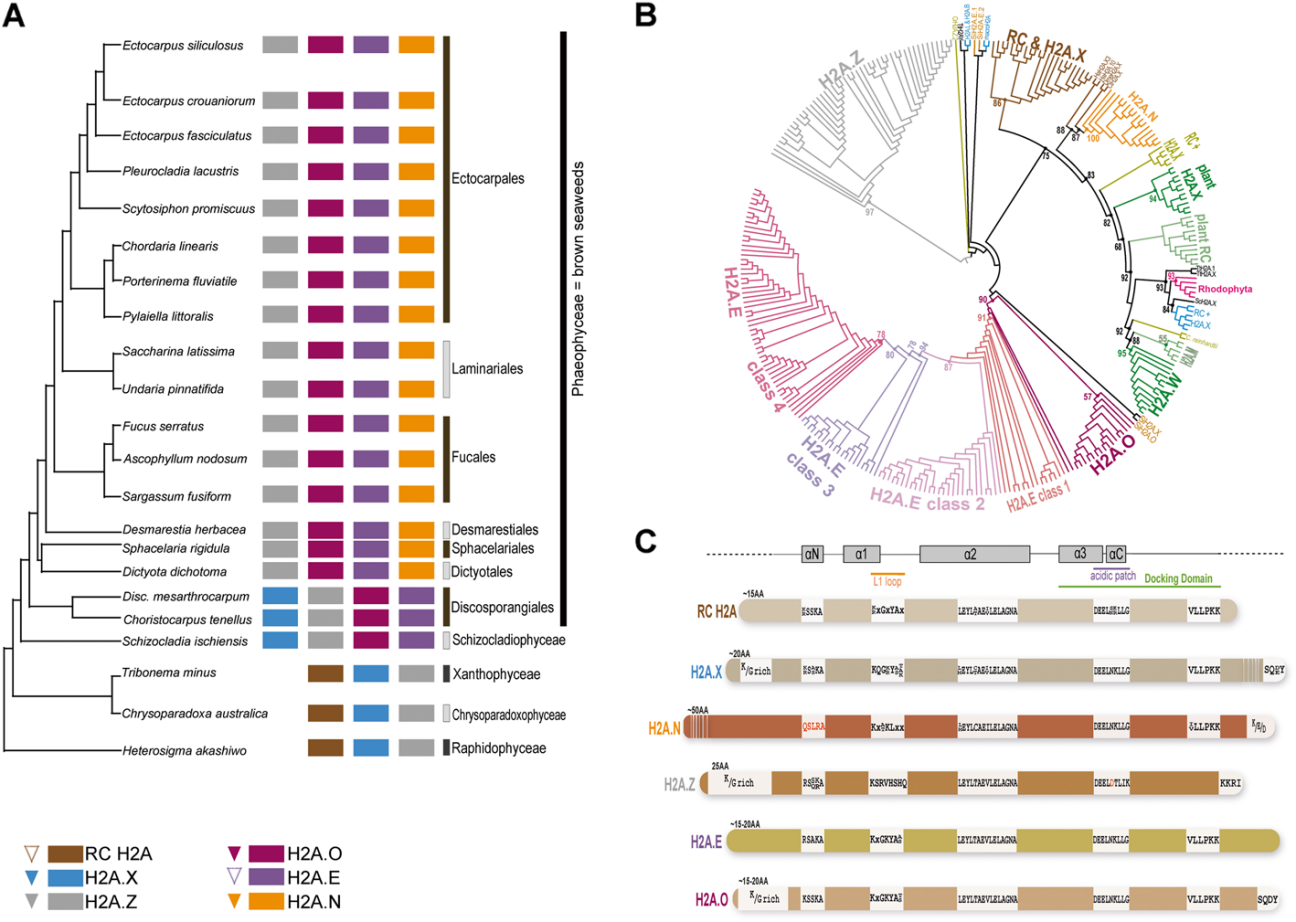
Identification of various H2A variants in brown seaweeds. **A** Schematic tree presenting the different H2A variants identified in brown seaweeds and sister taxa. This schematic tree was inferred from [17]. For each species from the Phaeoexplorer dataset, the presence of a H2A variant is indicated by a box. RC H2A, brown; H2A.X, blue; H2A.Z, grey; H2AO, purple; H2A.E, violet; H2A.N, orange. *Disc. mesarthrocarpum, Discosporangium mesarthrocarpum.* The presence of introns in different classes of H2A gene is depicted by triangles in the key. Introns are very rare in genes encoding either RC H2A or H2A.E (indicated by an empty dotted triangle). **B** Protein phylogeny based on maximum likelihood analysis for H2A variants from 18 brown seaweed species and four sister taxa along with representative species from yeast, Alveolates, animals, land plants, green and red algae. This phylogeny is represented as a circular cladogram with branch lengths unscaled to divergence (for a phylogram with branch lengths scaled to divergence, see Additional file 1: Fig. S1A). The H2A.E proteins group into four main classes: 1 to 4*.* Bootstrap values are indicated for key nodes. *Chlamydomonas reinhardtii* (Cr)*, Choristocarpus tenellus* (Ct), *Discosporangium mesarthrocarpum* (Dme), *Heterosigma akashiwo* (Ha)*, Saccharomyces cerevisiae* (Sc), *Schizocladia ischiensis* (Si), *Tetrahymena thermophila* (Tt). Plant, animal, green and red algal H2A proteins are displayed in dark green, blue, light green and pink respectively. For yeast and *T. thermophila*, they are displayed in black. The RC H2A and H2A.X proteins from Stramenopiles are displayed in brown. The H2A.Z proteins are displayed in grey. **C** Schematic representation of the different H2A variants identified in brown seaweeds and sister taxa. The consensus sequences for the αN helix, L1 loop, α2 helix and acidic patch are indicated. The motifs identified in the C-terminal tails of the H2A.X and H2A.Z and H2A.O variants are indicated. The presence of enriched amino acids (K/G rich) in N-terminal tails of H2A.X and H2A.Z and H2A.O variants is shown as well as in the C-terminal tail of the H2A.N variant (K/D/E). If present, the conserved sequence of the docking domain is indicated (mainly VLLPKK). The L1 loop, acidic patch and docking domain are underlined in orange, purple and green, respectively. The helices are indicated by grey rectangles. Black and red letters correspond to highly conserved or semi-conserved residues, respectively. The length of the N-terminal tail is indicated (AA, amino acids) for each H2A variant. With the exception of the H2A.N N-terminal and H2A.X C-terminal tails, which are much longer than shown, boxes are proportional to the protein size

In our previous study, we identified various histone proteins in brown algae and described H3 variants based on sequence similarity with mammalian proteins [17]. In order to identify and characterize H2A variants in brown algae, we analyzed a set of 22 reference genomes from the Phaeoexplorer database to identify all the predicted H2A proteins at both the transcript and the gene level (Additional file 2: Tables S1 & S2). These genomes belong to 18 species from seven different orders of the class Phaeophyceae (hereafter referred to as brown seaweeds) and four additional species (*Heterosigma akashiwo, Chrysoparadoxa australica, Schizocladia ischiensis* and *Tribonema minus*) were included as closely-related outgroup taxa (Fig. 1A). We also added data from *C. tenellus* to have a more comprehensive view of the evolutionary relationship between the brown algal orders Discosporangiales and Dictyotales. With the exception of *H. akashiwo* and *C. australica,* the chosen species are all multicellular. We performed phylogenetic analyses of identified H2A variants (Fig. 1B) encoded by these 22 Stramenopile genomes along with H2A sequences from representative species of Rhodophyta, Chlorophyta, land plants, animals and unicellular eukaryotes such as yeast (see Additional file 1: Fig. S1A for the phylogenetic relationships of the diverse eukaryotic species analyzed and Fig. 1A for phylogenetic relationships with the brown algae). As previously described [18], H2A.Z from the diverse species grouped into a monophyletic clade (Fig. 1B). The phylogeny in Fig. 1B is also consistent with several observations from previous studies: the H2A.M and H2A.W variants are likely to be derived from a common ancestor [19]; plant RC H2As group independently of H2A.X proteins [19]. In addition to known H2A variants, our analyses identified three novel distinct clades of H2A variants (H2A.N, H2A.O, H2A.E) with unique characteristics in Stramenopiles (Fig. 1C).

### RC H2A and H2A.X are absent from brown seaweeds

**Fig. 2:**
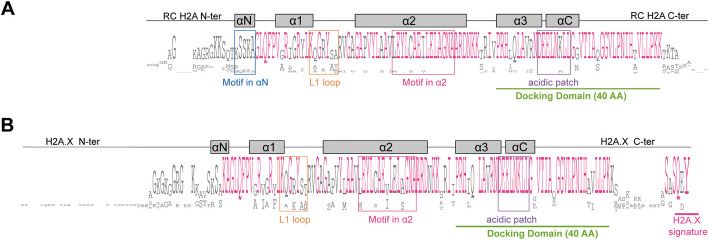
Characterization of RC H2A and H2A.X in brown seaweeds and sister taxa*.* **A**, **B** Consensus sequences for RC H2A (**A**) and H2A.X sequences (**B**) generated using Jalview [21], run with either the RC H2A sequences from *T. minus,* C. *australica* and *H. akashiwo* or with the H2A.X sequences from the brown seaweeds *C. tenellus* and D. *mesarthrocarpum* (both belong to the Discosporangiales) and the four sister taxa. The mature protein without the initial methionine is displayed. The docking domain is underlined in green. The length of the docking domain is indicated (AA, amino acids). The helices are indicated by grey rectangles. N-ter, N-terminal tail; C-ter, C-terminal tail. Regions used to generate logos of amino acid bias for the αN helix, L1 loop, α2 helix and acid patch are indicated by blue, orange, pink and purple squares, respectively. The corresponding logos are presented in Additional file 1: Fig. S2E-G for L1 loop, α2 helix and acid patch of the H2A.X variant and in Additional file 1: Fig. S3 for αN helix (Additional file 1: Fig.S3E middle panel), L1 loop (Additional file 1: Fig.S3F right panel), α2 helix (Additional file 1: Fig.S3G right panel) and acid patch (Additional file 1: Fig.S3H right panel) of the RC H2As

Within the Stramenopiles, we identified RC H2As (Fig. 1A) only in *H. akashiwo, C. australica* and *T. minus*, which are closely-related outgroups for the brown algae. The H2A.X variant was also detected in all three of the above species but also in *S. ischiensis* and in the Discosporangiales within the brown seaweeds (Fig. 1A). Note that classification of variants in this study was based on protein sequence signatures (Figs. 1C & 2A) and not on gene expression pattern during the cell cycle as the latter cannot currently be obtained for the brown algae.

The short branch-lengths for the RC H2As and H2A.X sequences from these species suggest slow rates of evolution (Additional file 1: Fig. S1A & S2B). The RC H2A and H2A.X proteins from *H. akashiwo, C. australica*, *T. minus* and diatoms grouped together in the same clade (with the exception of HaH2A.10 and HaH2A.X.3), with high bootstrap support (Fig. 1B, Additional file 1: Fig. S2B). Grouping of proteins within the RC H2A and H2A.X clade reflected species phylogeny [20], suggesting concerted evolution of RC H2A and H2A.X genes within each species. This feature has not been reported in plants where RC H2A and H2A.X constitute independent clades (Fig. 1B). Finally, we found that the *D. mesarthrocarpum* and *C. tenellus* H2A.X variants have longer N-terminal tails than those of other species (Additional file 1: Fig. S2C). *D. mesarthrocarpum* and *C. tenellus* H2A.X variants seem to share a common ancestry with H2A.N (Fig. 1B, Additional file 1: Fig. S2B). Regarding sequence characteristics, the C-terminal tails of the RC H2A proteins of *H. akashiwo, C. australica* and *T. minus* show lysine enrichment (Fig. 2A, Additional file 1: Fig. S2A). Plant and animal RC H2As also have C-terminal tails enriched in acidic residues (Additional file 1: Fig. S2B).

### Most brown seaweeds possess the novel H2A.N variant

**Fig. 3:**
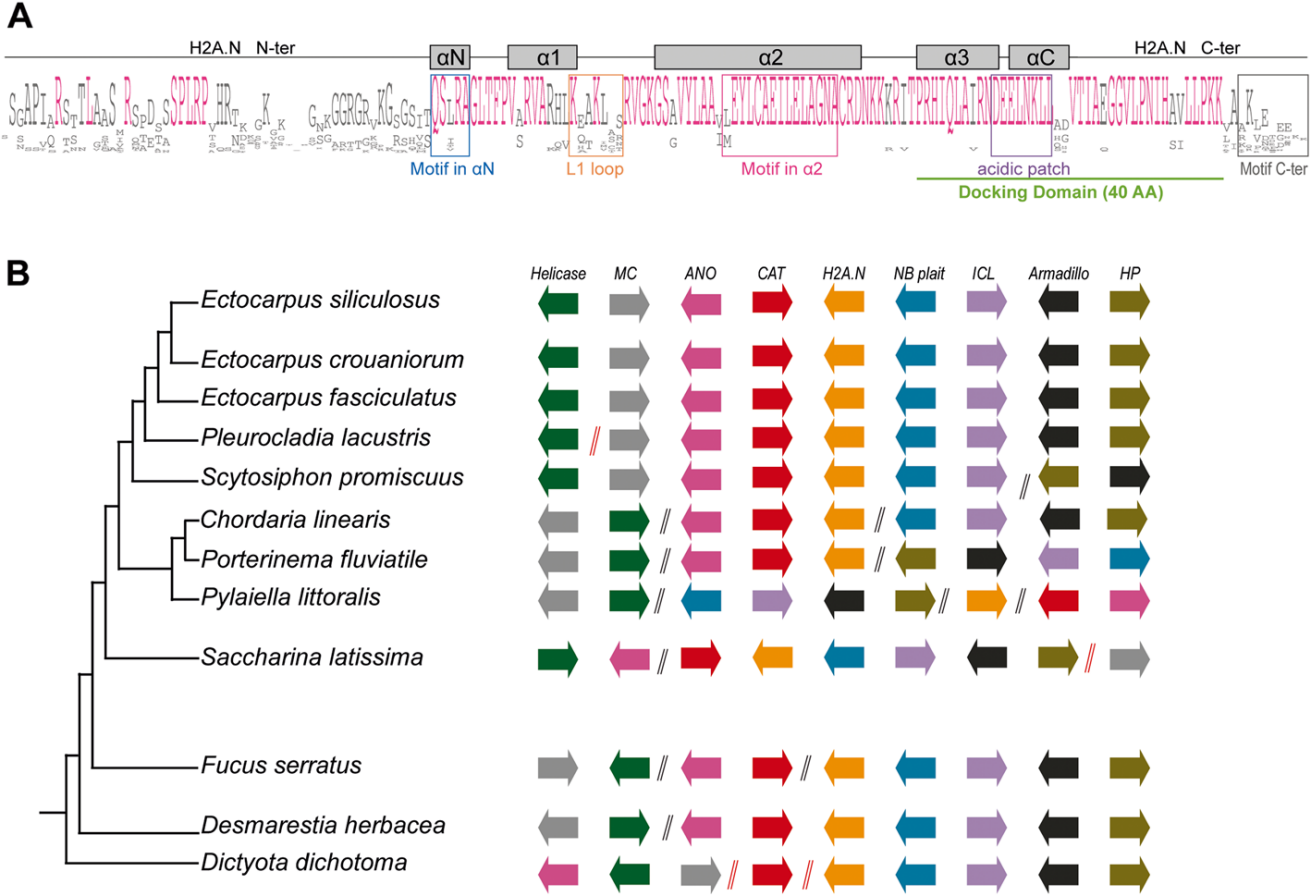
Characterization of the H2A.N variants in brown seaweeds. **A** Consensus sequences for H2A.N sequences generated using Jalview [21], run with the H2A.N sequences from 16 brown seaweed species (all Phaeophyceae, with the exception of the two Discosporangiales species, *C. tenellus* and *D. mesarthrocarpum,* that do not have the H2A.N variant). The mature protein without the initial methionine is displayed. The docking domain is underlined in green. The length of the docking domain is indicated (AA, amino acids). The helices are indicated by grey rectangles. N-ter, N-terminal tail; C-ter, C-terminal tail. Regions used to generate logos of amino acid bias for the αN helix, L1 loop, α2 helix, acid patch and C-terminal tail are indicated by blue, orange, pink, purple and grey squares, respectively. The corresponding logos are presented in Additional file 1: Fig. S3 for αN helix (Additional file 1: Fig. S3E left panel), L1 loop (Additional file 1: Fig. S3F left panel), α2 helix (Additional file 1: Fig. S3G left panel), acid patch (Additional file 1: Fig. S3H left panel) and C-terminal tail (Additional file 1: Fig. S3I) of the H2A.N variant. **B** Schematic representation of the genomic neighborhood showing shared synteny for the H2A.N variant. The genomic region represents the four genes located on both sides of the *H2A.N* gene, along with a phylogenetic tree of species used for the synteny analysis. Each gene is displayed as an arrow to indicate gene orientation. Double slashes indicate breaks in synteny (black double slashes indicate that some additional genes are intercalated while red double slashes indicate that genes downstream of this symbol are located on another contig). The *H2A.N* gene is represented in orange and each other gene by a different color. We named genes according to their description in JBrowse: Helicase, C-terminal, Helicase; Mitochondrial Carrier, MC; Anoctamin, ANO; Carnitine/choline O-acyltransferase, CAT; Nucleotide-binding alpha-beta plait domain, NB plait; Isochorismatase-like, ICL; Armadillo-like helical, Armadillo; hypothetical protein, HP

Brown seaweeds (with the exception of *D. mesarthrocarpum* and *C. tenellus*, Fig. 1A) possess proteins with a longer N-terminal tail compared to other H2A variants (Fig. 1C), extended by approximately 30 amino-acids (Fig. 3A). Since these H2A variants have a long N-terminal tail, we named them H2A.N, following the histone nomenclature established by [18]. Our phylogenetic analysis showed that all the H2A.N proteins grouped into a single phylogenetic clade, which clustered with two RC H2A proteins from *H. akashiwo* and H2A.X from Discoporangiales (Fig. 1B, Additional file 1: Fig. S3A). We then performed a closer inspection of their sequence features. AlphaFold [22, 23] did not produce a trustworthy structure prediction for the unusually long N-terminal tail. In addition to its unique N-terminal tail, H2A.N has a highly divergent motif in its αN helix (QSLRA, Fig. 1C & Additional file 1: Fig. S3E) but with conserved predicted helix structure. The H2A.N variant and RC H2A histones have similar features (Additional file 1: Fig. S3F-H). The C-terminal tails of H2A.N proteins are enriched in lysines and acidic residues (Fig. 3A, Additional file 1: Fig. S3I) as are the C-terminal tails of plant and animal RC H2As (Additional file 1: Fig. S2B).

Furthermore, we performed a synteny analysis to identify *H2A.N* orthologues in the genomes of sister taxa species of brown seaweeds and investigate whether H2A.N is orthologous to RC H2A. Despite several minor genomic rearrangements, there was a clear shared synteny between the genomic regions containing the *H2A.N* gene across brown seaweeds (Fig. 3B). In contrast, we noticed that the genes surrounding the *H2A.N* gene in brown algae are dispersed in the genome of *S. ischiensis*, which lacks H2A.N. Furthermore, while RC H2A genes are located on contigs that contain additional histone-coding genes in most organisms and more specifically in *H. akashiwo* (Additional file 2: Table S7), *H2A.N* genes are localized on contigs without such genes (Additional file 2: Table S3). Therefore, although H2A.N and RC H2A share some characteristics, H2A.N does not seem to be orthologous to RC H2A.

### Brown seaweeds two possess novel H2A variants with a H2A.Z signature: H2A.E and H2A.O

**Fig. 4:**
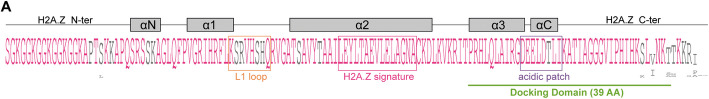
Characterization of the H2A.Z variant in brown seaweeds*.* **A** Consensus sequences for H2A.Z proteins generated using Jalview [21] run with the H2A.Z sequences from the 18 brown seaweed species and the four sister taxa. The mature protein without the initial methionine is displayed. The H2A.Z signature in the α2 helix is indicated with a pink square. The docking domain is underlined in green. The length of the docking domain is indicated (AA, amino acids). The helices are indicated by grey rectangles. N-ter, N-terminal tail; C-ter, C-terminal tail. Regions used to generate logos of amino acid bias for the L1 loop and acid patch are indicated by orange and purple squares, respectively. The corresponding logos are presented in Additional file 1: Fig. S4 for L1 loop (Additional file 1: Fig. S4B) and acid patch (Additional file 1: Fig. S4C) of the H2A.Z variant

**Fig. 5:**
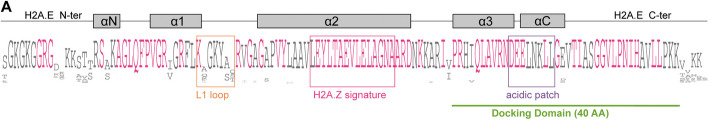
Characterization of the H2A.E variant in brown seaweeds*.* **A** Consensus sequences for H2A.E proteins generated using Jalview [21] run with the H2A.E sequences from the 18 brown seaweed species and the sister species *S. ischiensis*. The mature protein without the initial methionine is displayed. The docking domain is underlined in green. The length of the docking domain is indicated (AA, amino acids). The helices are indicated by grey rectangles. N-ter, N-terminal tail; C-ter, C-terminal tail. Regions used to generate logos of amino acid bias for the L1 loop and acid patch are indicated by orange and purple squares, respectively. The corresponding logos are presented in Additional file 1: Fig. S5 for L1 loop (Additional file 1: Fig. S5D) and acid patch (Additional file 1: Fig. S5E) of the H2A.E variant

**Fig. 6:**
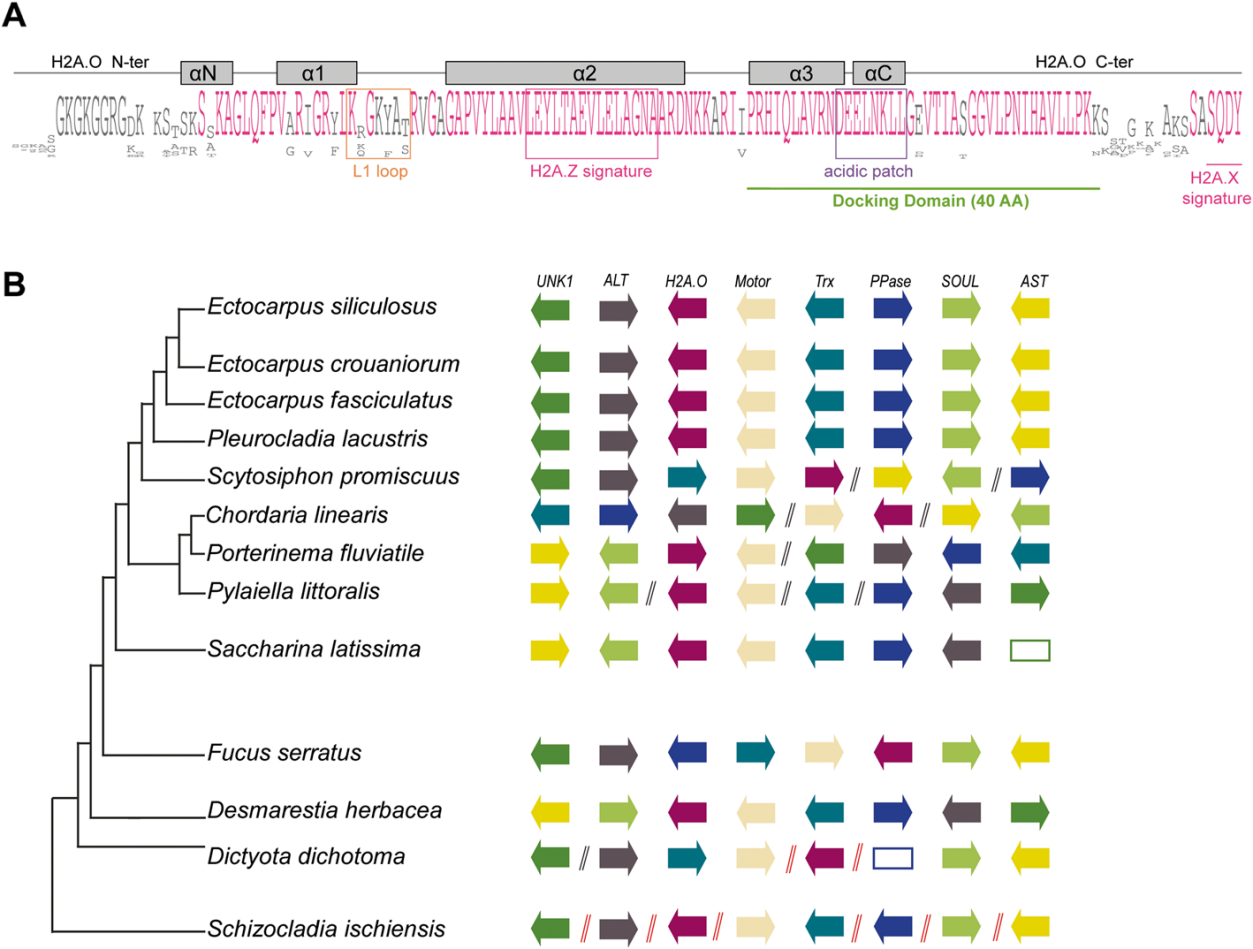
Characterization of the H2A.O variant in brown seaweeds*.* **A** Consensus sequences for H2A.O proteins generated using Jalview [21] run with the H2A.O sequences from the 18 brown seaweed species and the sister species *S. ischiensis*. The mature protein without the initial methionine is displayed. The docking domain is underlined in green. The length of the docking domain is indicated (AA, amino acids). The helices are indicated by grey rectangles. N-ter, N-terminal tail; C-ter, C-terminal tail. Regions used to generate logos of amino acid bias for the L1 loop and acid patch are indicated by orange and purple squares, respectively. The corresponding logos are presented in Additional file 1: Fig. S6 for L1 loop (Additional file 1: Fig. S6B) and acid patch (Additional file 1: Fig. S6C) of the H2A.O variant. **B** Schematic representation of the genomic neighborhood showing shared synteny for the H2A.O variant. The genomic region represents the two genes located upstream of the *H2A.O* gene and four genes downstream, along with a phylogenetic tree of species used for the synteny analysis. Each gene is displayed as an arrow to indicate gene orientation. Double slashes indicate breaks in synteny (black double slashes indicates that some additional genes are intercalated while red double slashes indicate that genes downstream of this sign are located on another contig). The *H2A.O* gene is represented in purple and each other gene by a different color. We named genes according to their description in JBrowse: conserved unknown protein, UNK1; alanine transaminase, ALT; kinesis-like protein, Motor; Thioredoxin-like protein, Trx; NTP pyrophosphohydrolase MazG putative catalytic core, PPase; SOUL haem-binding protein, SOUL; aspartate aminotransferase, AST. The *UNK1* and *PPase* genes were absent from *S. latissima* and *D. dichotoma*, respectively. These missing genes are thus represented as empty rectangles

For each protein identified with a H2A.Z signature in the α2 helix [8], we further analyzed motifs present in the L1 loop, N- and C-terminal tails and combined this information with the results from the phylogenetic analysis (Fig. 1B). This approach identified three different H2A variants: H2A.Z (Fig. 4A) plus two novel variants H2A.E (Fig. 5A) and H2A.O (Fig. 6A), both named following the histone nomenclature established by [18]. The H2A.Z proteins from brown seaweeds and closely-related sister group species were found as a single copy in all the studied Stramenopile species (Additional file 2: Table S4) and these proteins clustered as a monophyletic group in the phylogenetic analysis (Fig. 1B, Additional file 1: Fig. S4A). Species phylogeny was well supported within this phylogenetic group (*e.g.* independent clades for Rhodophyta, Chlorophyta, land plants or animals, Additional file 1: Fig. S4A). Brown seaweed H2A.Z variants had H2A.Z-specific features (Fig. 4A, Additional file 1: Fig. S4B-D) along with a long lysine- and glycine-rich stretch in their N-terminal tails (Figs. 1C & 4A).

The brown algal H2A.E and H2A.O variants formed a single monophyletic clade with high bootstrap support (Fig. 1B, Additional file 1: Fig. S1B). They are present both in *S. ischiensis* and in the brown seaweeds (Fig. 1A). H2A.E and H2A.O differ by a long stretch of lysine and glycine in the N-terminal tail and a highly conserved SQDY signature in the C-terminal tail, both features being found only in the H2A.O variant (Fig. 1C, Fig.6A).

We identified four main phylogenetic clades for the H2A.E variants (Fig. 1B, Additional file 2: Table S5): proteins from the four classes differ in their αN helix (Additional file 1: Fig. S5F) and L1 loop consensus (Additional file 1: Fig. S5). H2A.O was encoded by a single gene in brown seaweed species but we identified several H2A.E variants for each investigated species (Additional file 2: Table S4). The H2A.E genes are associated in tandem with H2B genes and co-localize with genes encoding H1, H3.1 and H4 (Additional file 2: Table S6). Note that RC H2A genes tend to be clustered with other RC histone genes and present in multiple copies (Talbert and Henikoff, 2010). The genomic neighborhood of H2A.E genes (*i.e.* occurrence in histone gene clusters) is thus similar to that of RC H2A genes (including for RC H2A genes in the Stramenopile *H. akashiwo*). A shared ancestry between RC H2A and H2A.E is thus very likely.

The H2A.O proteins are phylogenetically clustered (Fig. 1B and Additional file 1: Fig. S6A). Since H2A.X and H2A.O variants share a SQ_[D/E]_Y signature in their C-terminal tail, we used synteny to investigate if they share a common ancestry. The genomic region containing the *H2A.O* gene was syntenic in brown seaweeds, with some minor rearrangements in the gene order (Fig. 6B). The genes that make up this syntenic cluster are dispersed in the *S. ischiensis* and *H. akashiwo* genomes (note that *S. ischiensis* has H2A.O but *H. akashiwo* does not). Moreover, H2A.X variant genes in *H. akashiwo* are not located in the vicinity of the orthologues of genes of the brown algal H2A.O syntenic cluster (Additional file 2: Table S7). We thus concluded that H2A.O is not the orthologue of H2A.X in brown seaweeds, despite the fact that the two variant classes share the SQ_[D/E]_Y signature.

### The H2A.E and H2A.O variants were found in various algae species

We identified three novel H2A variants in brown seaweeds, H2A.N, H2A.E and H2A.O (Fig. 1C). This prompted us to explore whether such variants exist in other algae. We searched in the genomes of other Stramenopiles *i.e.* pennate diatoms (*Phaeodactylum tricornutum, Fragilariopsis cylindrus)* as well as in green (the unicellular species *Ostreococcus tauri, Ostreococcus lucimarinus, Chlamydomonas reinhardtii*) and red algae (the multicellular species *Chondrus crispus* and the unicellular species *Galdieria sulphuraria, Porphyridium purpureum* and *Cyanidioschyzon merolae*). We also analyzed a recently released dataset of deep genomics from multicellular red and green algae [24]. We did not detect H2A.N variants in the investigated genomes but we identified H2A.E variants in two green and two red algal species and a H2A.O variant in one red algal species.

**Fig. 7:**
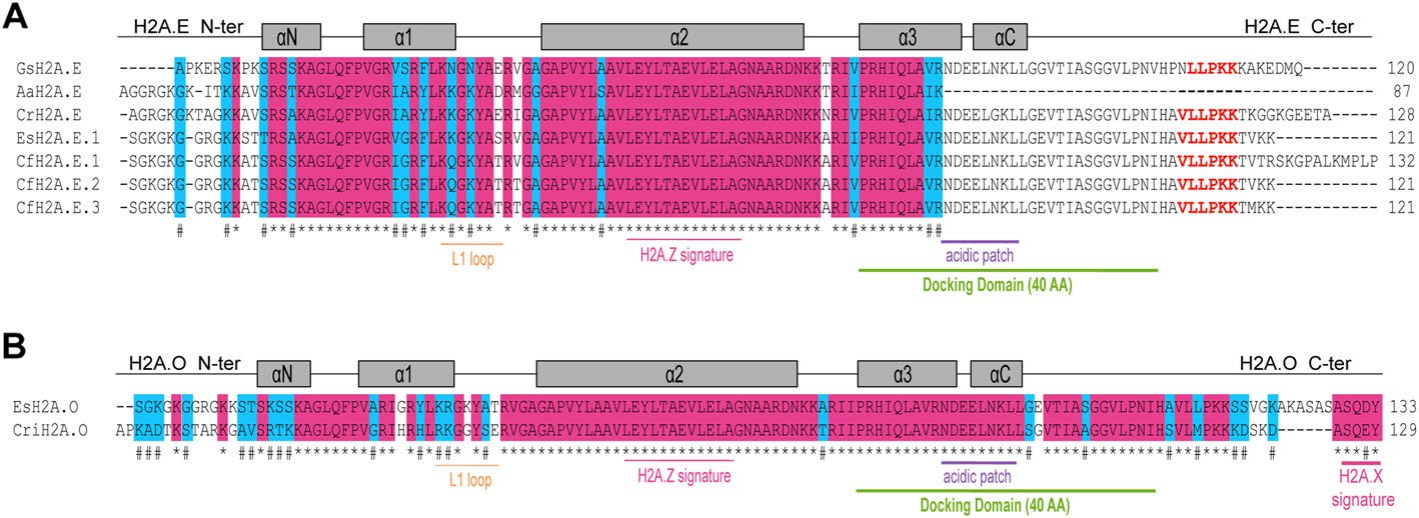
Identification of the H2A.E and H2A.O variants in green and red algae*.* **A**, **B** Alignment of the H2A.E (**A**) and H2A.O (**B**) proteins identified in green and red algae. The L1 loop, acidic patch and docking domain are underlined in orange, purple and green, respectively. The H2A.Z signature in the α2 helix is indicated and underlined in pink for the H2A.E and H2A.O variants. The H2A.X signature in the C-terminal tail of the H2A.O variant is indicated and underlined in pink. The length of the docking domain is indicated (AA, amino acids). The helices are indicated by grey rectangles. Asterisks indicate a fully conserved residue and the hash conservation between residues with either strong or weak similar properties. *Avrainvillea amadelpha* (Aa), *Catenella fusiformis* (Cf), *Chlamydomonas reinhardtii* (Cr)*, Chroothece richterianum* (Cri), *Ectocarpus siliculosus* (Es), *Galdieria sulphuraria* (Gs)

In green algae, we identified a H2A.E variant in the unicellular species *C. reinhardtii* (CrH2A.E, Fig. 7A). Phylogenetic analysis showed that RC H2A and H2A.E from *C. reinhardtii* constituted a clade that was distinct from other H2A.E proteins and the former clustered with the H2A.M and H2AW variants (Additional file 1: Fig. S1B). Furthermore, we identified a H2A.E variant in the multicellular species *Avrainvillea amadelpha* (AaH2A.E, Fig. 7A). In red algae, the unicellular species *G. sulphuraria* has only a single H2A protein, which is a H2A.E variant (GsH2A.E, Fig. 7A). This protein belongs to the Rhodophyta H2A clade (Additional file 1: Fig. S1B). Additionally, we identified three H2A.E variants in the multicellular species *Catenella fusiformis* (Fig. 7A), and a H2A.O variant in *Chroothece richterianum,* another multicellular species (Fig. 7B). To conclude, we identified H2A.E and H2A.O variants in species from both the green and red algal lineages.

### Intron structure and organization of histone genes in brown algal genomes

Genes encoding RC histones tend to be clustered while those coding for RI variants are more dispersed within animal genomes [25]. In the present study, we identified the gene encoding each H2A variant (Additional file 2: Table S2) and analyzed its genomic location (Additional file 2: Table S3). We also analyzed the number of introns in each H2A gene (Additional file 2: Table S2). We excluded *Sargassum fusiform* and *Tribonema minus* from this analysis since their genome assemblies were not of sufficient quality to identify extensive gene clusters.

The sister group species closely-related to brown seaweeds, *H. akashiwo* and *C. australica,* have several RC H2A proteins, each of which is encoded by a single gene (Additional file 2: Table S2). Most of these genes have no introns and, as reported in mammals [26], they are located on contigs that contain other histone-coding genes (Additional file 2: Table S3). In brown seaweeds, the H2A.Z variant is encoded by a single gene that has introns. These H2A.Z genes are frequently localized on contigs that contains other histone genes. The H2A.N and H2A.O variants are also encoded by single genes (Additional file 2: Table S2), but these are localized on contigs that do not contain additional histone genes (Additional file 2: Table S3). *H2A.O* genes have one intron in most cases but *H2A.N* genes and the *H2A.X* genes in *H. akashiwo* and *C. australica* have several introns*.* Therefore, as was also observed in mammals [25, 26], genes coding RI variants contain introns (Fig. 1A) and are dispersed in brown seaweed genomes.

For the H2A.E variant, each protein was encoded by several genes in most cases, (Additional file 2: Table S2) and these genes were either dispersed across the genome or localized in tandem arrays or in close vicinity. The *H2A.E* genes co-localised with genes encoding H1, H2B, the RC H3.1 variant and H4 (Additional file 2: Table S3), confirming the presence of histone gene clusters in brown seaweeds. Indeed, in *Ectocarpus* species 7 (hereafter referred as *E.* sp7), histone genes were found to be organized into clusters [14]. Mammalian RC H2A genes do not have introns [26] and this is also the case for most *H2A.E* genes (Additional file 2: Table S2, Fig. 1A). The presence of *H2A.E* genes in histone gene clusters as in the case of RC H2A genes, as well as the presence of several H2A.E variants and the absence of introns, support the hypothesis of a shared ancestry with RC H2A, as mentioned above.

### Different algal H2A variants undergo distinct PTMs

**Fig. 8:**
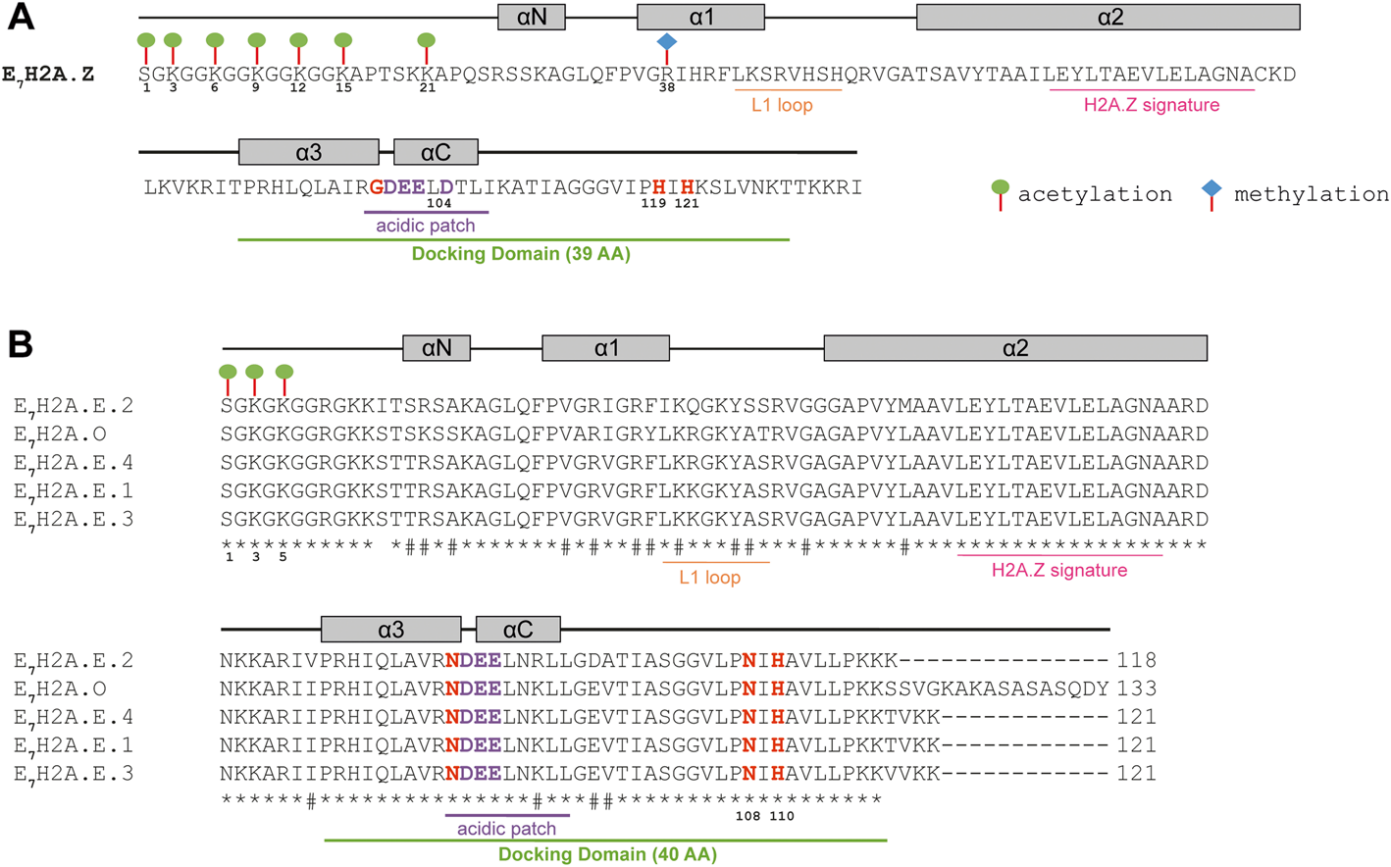
Predicted presence of PTMs on H2A variants from *Ectocarpus* species 7*.* **A** Protein sequence of the H2A.Z variant from *Ectocarpus* species 7*.* The two histidine residues present only in docking domains of H2A.Z proteins are displayed in red. Acidic patches start with a glycine in H2A.Z (displayed in red). The L1 loop, acidic patch, docking domain and the H2A.X signature and the H2A.Z signature in the α2 helix are underlined in orange, purple, green and pink, respectively. The length of the docking domain is indicated (AA, amino acids). The helices are indicated by grey rectangles. PTMs are shown based on data from [14]. **B** Alignment of *Ectocarpus* species 7 H2A variants. In contrast to H2A.Z, the H2A.E and H2A.O variants harbor only one histidine residue (position 110, in red) in their docking domains, the first one is replaced by an asparagine (position 108, in red). Acidic patches start with an asparagine (displayed in red). Asterisks indicate fully conserved residues and hashes indicate conservation between residues with either strong or weak similar properties. PTMs are shown based on data from [14]

Histones are marked by a great variety of post-translational modifications. A mass spectrometry approach has been applied to *E.* sp7 to characterize histone PTMs [14]. We inspected the predicted peptides to assign these modifications to the corresponding H2A variants identified in the present study. The N-terminal tail of the *E.* sp7 H2A.Z is enriched in acetylated lysine residues (K3, K6, K9, K12, K15, K21) and has an acetylated S1 and a methylated R38 (Fig. 8A) [14]. The N-terminal tail of diatom H2A.Z is also enriched in lysine residues and has been reported to be acetylated at S1/K3/K6/K9/K12/K15 but neither K21 acetylation nor R38 methylation was detected [12]. Both serine and lysine acetylation (S1, K3, K5) have been reported for another peptide [14] that occurs in all the H2A.E variants and the H2A.O variant from *E.* sp7 (Fig. 8B). We also investigated putative phosphorylation sites in the different H2A variants from *E.* sp7*,* an aspect not investigated in the previous study [14]. The different *E. siliculosus* H2A variants have residues at several positions along the protein that could potentially undergo phosphorylation, with differences between variants (Additional file 1: Fig. S8). Therefore, amino acid sequence differences between H2A variants potentially impact PTM deposition.

### H2A variants were differentially expressed in reproductive tissues and in response to environmental conditions

Some histone variants have been reported to have sperm-specific expression patterns in *Arabidopsis* [27] or testis-specific expression patterns in mammals [28]. These variants can also be involved in development or stress responses (reviewed in [29]). Since the functions of the novel algal H2A variants in these various mechanisms remain elusive, we first evaluated expression of histone genes in various vegetative and reproductive tissues and under different growth conditions. We used publically available RNA-Seq data (i) from vegetative and reproductive tissues of *Desmarestia herbacea, Dictyota dichotoma, Fucus serratus* and *E.* sp7 and (ii) from *Pleurocladia lacustris* and *Porterinema fluviatile* under two different experimental water conditions. Generally, expression of *H2A.Z, H2A.O, H2A.N* genes is ubiquitous. Conversely, not all H2A.E genes are expressed and some genes are specifically expressed in sperm cells.

**Fig. 9:**
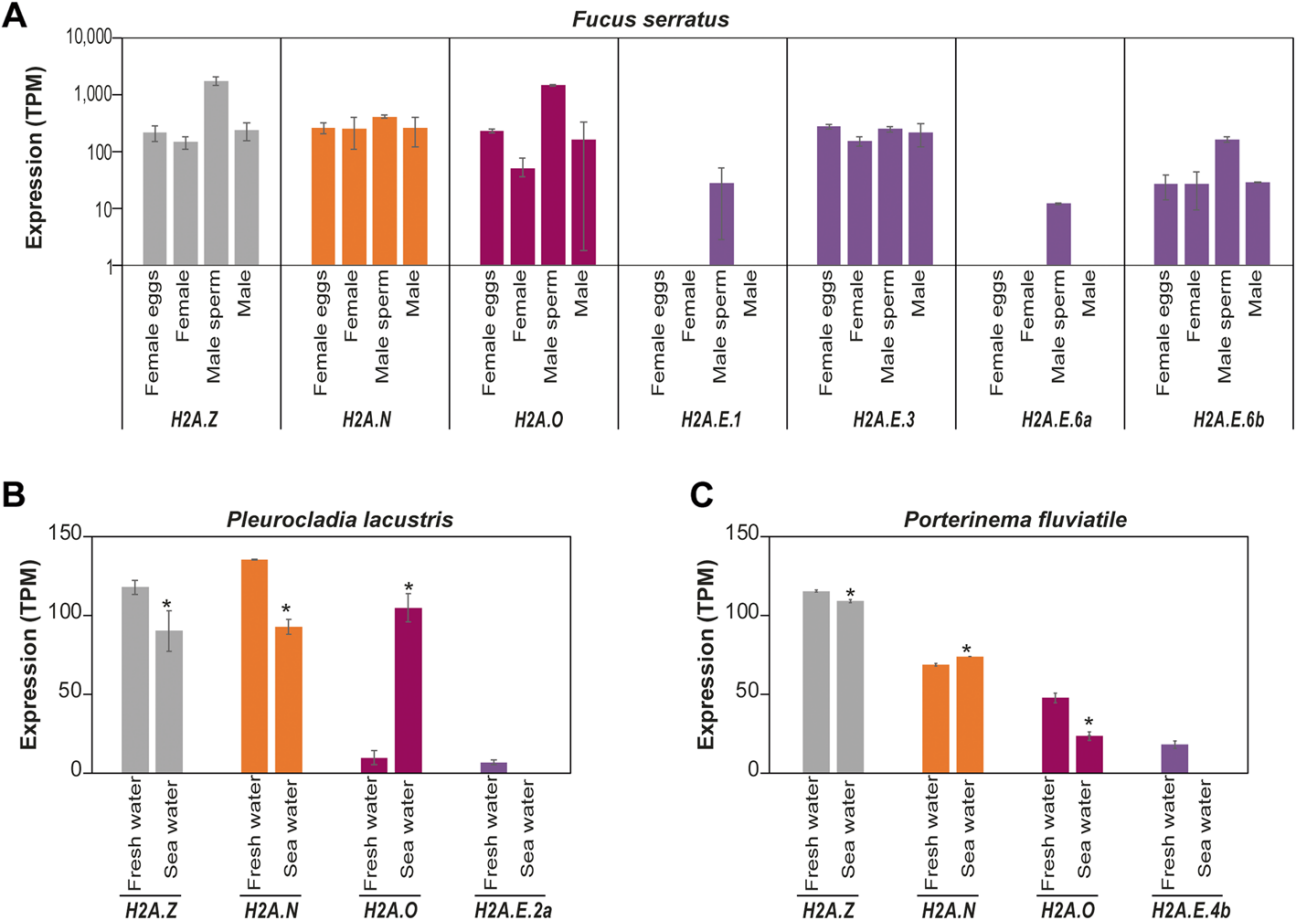
Analysis of the gene expression for the different H2A variants*.* **A** Expression of genes encoding *F. serratus* H2A variants. The histogram represents the mean transcript abundance in TPM (Transcripts Per Kilobase Million) displayed with a logarithmic scale. It corresponds to RNA-Seq data obtained from three biological replicates consisting of independent cultures of female and male gametophyte thalli (referred to as female and male, respectively) as well as from released female eggs and male sperm. Only genes with a TPM value above 2 are displayed. **B**, **C** Expression of the genes coding the *P. lacustris* (b) and *P. fluviatile* (c) H2A variants. The histogram represents the mean transcript abundance in TPM (Transcripts Per Kilobase Million). It corresponds to RNA-Seq data obtained from three biological replicates consisting of independent cultures grown either in fresh or sea-water. Only genes with a TPM value above 2 for at least one condition are displayed. Student’s t test; * *P* < 0.05. Each H2A variant is depicted in a different color with a code similar to that used in Fig. 1A

With regard to tissue-specific expression, in *D. dichotoma* (Additional file 1: Fig. S9C), *F. serratus* (Fig. 9A) and *E.* sp7 (Additional file 1: Fig. S9E), the *H2A.Z, H2A.O* and *H2A.N* genes displayed ubiquitous expression across different life cycle stages though at varying levels (Additional file 1: Fig. S9C). In *D. herbacea,* all the *H2A.E* genes were expressed, with particularly high transcript levels in male sperm (Additional file 1: Fig. S9D). However, in *F. serratus* (Fig. 9A) and *E.* sp7 (Additional file 1: Fig. S9E), only some of the *H2A.E* genes were expressed and the expression of these genes was detected either in all samples or only in male sperm. This analysis suggested that the H2A.E.1 variant of *F. serratus* and the H2A.E.1 and H2A.E.2 variants of *E.* sp7 could be sperm-specific variants.

To investigate if the diverse H2A variants could be involved in environmental responses, we compared gene expression in *P. lacustris* and *P. fluviatile* under two different experimental conditions: freshwater and seawater. In both species, the *H2A*.Z transcripts were more abundant in freshwater than in seawater (Fig. 9B-C). However, the expression patterns of *H2A.N* and *H2A.O* differed in the two species under the freshwater and seawater conditions, suggesting that there are also species-specific differences (Fig. 9B-C). For both species, we detected the expression of only one H2A.E gene that was exclusively expressed in freshwater conditions (*P. lacustris H2A.E.2a* and *P. fluviatile H2A.E.4b*, Fig. 9B-C). Hence, expression of genes encoding brown seaweed H2A.Z, H2A.N and H2A.O appeared ubiquitous under various environmental conditions while most *H2A.E* genes were not expressed.

## Discussion

In this study, we carried out a comprehensive analysis to identify H2A variants in brown seaweeds and sister taxa species, together with an analysis of available green and red algal genomes. Our study relied on phylogenetic analysis combined with analysis of seven protein features (see Methods). This approach enabled us to identify the different variants present in brown seaweeds and to define their signatures (Fig. 1C). In addition to H2A.X and H2A.Z, which are both widespread eukaryotic variants, we report novel H2A variants present either in three (H2A.E: Rhodophyta, Chlorophyta and Stramenopiles) or two (H2A.O: Rhodophyta and Stramenopiles) algal clades. We also identified a brown-algae-specific variant, H2A.N, that is present in most brown seaweed orders. Our phylogenetic analysis revealed that, compared to the short variants H2A.B or H2A.L, which had long branch lengths in the phylogenetic tree (Additional file 1: Fig. S1B) consistent with a previous report [28], the novel algal H2A variants exhibited short branch lengths, suggesting slow rates of evolution. We thus hypothesize that they have evolved more slowly than the mammalian short H2A variants.

The H2A.E variant was not only found in brown seaweeds but also in two other independent lineages, Chlorophyta and Rhodophyta. The brown algal H2A.E and H2A.O variants arose from a single monophyletic clade but phylogenetic analysis indicated that H2A.E proteins from the three algal clades (brown, red and green) were not monophyletic. Consequently, we hypothesize that the H2A.E variants evolved several times independently, as proposed for the RC H2A and H2A.X variants [18]. Additionally, we observed that H2A.E genes are localized in tandem with H2B genes in histone gene clusters containing other genes encoding H2A.Z, H1, the RC H3.1 variant and H4. The H2A.E genes are also intron-less in most cases. Both features are also characteristic of RC H2As in mammalian genomes [25, 26]. Altogether, these data strongly suggested that RC H2A and H2A.E share a common ancestry in brown algae. Moreover, the number of H2A.E proteins has expanded dramatically in brown seaweeds, which are multicellular organisms. The unicellular green alga *C. reinhardtii*, which has a genome of a similar size to that of many brown seaweeds, possesses only one H2A.E protein. Moreover, in the red lineage, the multicellular alga *C. fusiformis* has three H2A.E proteins while the unicellular species *G. sulphuraria* has only one H2A.E protein. Thus, H2A.E expansion appears to be linked to multicellularity rather than to genome size.

Brown seaweeds lack RC H2A but possess the H2A.N and H2A.E variants. The *H2A.E* genes are expressed with tissue-specific patterns but are present within histone gene clusters, whereas expression of *H2A.N* is ubiquitous (Fig. 9 and Additional file 1: Fig. S9). The H2A.N variant also shares some protein features with RC H2A (a C-terminal tail enriched in lysine and acidic residues). Presence of several RC histone gene copies along with genomic clustering enable the production of large amounts of histones during the S-phase [30]. Therefore, based on their genomic location and protein features, it is thus possible that H2A.E and H2A.N may, respectively, either participate to the elevated histone transcription during DNA replication or perform functions similar to those done by RC H2A in other lineages, although the functional specialization of RC H2A has yet to be determined. These hypotheses regarding the putative roles of H2A.N and H2A.O variants require experimental validation.

Regarding H2A.O, this variant was found not only in brown seaweeds and Schizocladiophyceae but also in the red seaweed *C. richterianum*. Therefore, either this variant evolved at least twice in algae or brown algae acquired the gene through ancestral endosymbiosis of a red alga that already possessed the H2A.O variant. Under the second hypothesis, several Ochrophyta lineages would have then lost this variant during their evolutionary history. Concomitant with the acquisition of H2A.O, brown seaweeds (with the exception of Discosporangiales) lost the H2A.X variant. Our synteny analysis revealed that a common ancestry between the H2A.X and H2A.O variants is unlikely. Therefore, during evolution, RC H2A might have diverged in sequence to give rise to the ancestor of the H2A.E and H2A.O variants. Then, the variant H2A.O variant might have diverged from H2A.E by the acquisition of a H2A.X-like C-terminal tail, these events being accompanied by loss of RC H2A and H2A.X during the emergence of the class Phaeophyceae. A similar scenario has been suggested for the *Drosophila* H2A.V variant [31].

All H2A.N variants have αN helices that share a strongly divergent QSLRA motif, suggesting that the emergence of this specific helix was likely to be the first event that led to the appearance of the H2A.N variant. A H2A protein (KAJ3710985.1) from the fungus *Lentinula raphanica* contains the same αN motif but lacks a long N-terminal tail. Moreover, the N-terminal tail of H2A.N has a PLRP motif (Additional file 1: Fig. S3D, brown square). This motif was reported in the human E3 protein ligase and is characterized by a proline stack and a loop conformation critical for the catalytic function of this enzyme [32]. The roles of the PLRP and QSLRA αN motifs in H2A.N remain to be investigated.

In the nucleosome, the H2A C-terminal tail interacts with DNA and protrudes from the nucleosome and this might enable interactions with various proteins, leading to H2A specialization. Hence, we speculate that the H2A.Z and H2A.N C-terminal motifs (KKRI and K/E/D, respectively) could be involved in protein binding, in order to control chromatin remodelling for example. Moreover, the H2A.Z acidic patch begins with a glycine (Fig. 4A) whereas it begins with an asparagine in all the other variants. This sequence variation could destabilize the H2A.Z/H3 interaction while the additional acidic residue in the docking domain might stabilize H2A.Z binding to the H4 N-terminal tail [33]. Therefore, the different features observed in H2A variant sequences are likely to impact nucleosome stability and chromatin structure. RC H2A and the H2A.X, H2A.O and H2A.E variants are expected to behave similarly in terms of nucleosome stability since they have a similar lengths and L1 loops.

Histones are decorated by various post-translational modifications that modulate genome expression. Several PTMs were previously identified on E. sp7 H2As by mass spectrometry, but without variant-specific resolution [14]. We reassigned these modifications to the corresponding H2A variants identified in the present study. However, their genomic distribution and abundance remain to be investigated. The H2A.Z protein has several lysines in its N-terminal tail that are targets for acetylation (Fig. 8A) [14]. This hyper-acetylation might destabilize the nucleosome [34]. Post-translational modifications can also be involved in signaling. Indeed, during double-strand DNA break repair, serine and tyrosine residues in the H2A.X SQ[D/E]Y motif are phosphorylated, as is the upstream threonine residue [35]. In this study, we report the presence of one or two phosphorylatable residues in H2A.X (Additional file 1: Fig. S2D) and H2A.O, respectively (Additional file 1: Fig. S8), and the N-terminal tail of H2A.N was enriched in phosphorylatable serines (Additional file 1: Fig. S8). These phosphorylatable residues represent potential sites for chromatin regulation.

**Fig. 10:**
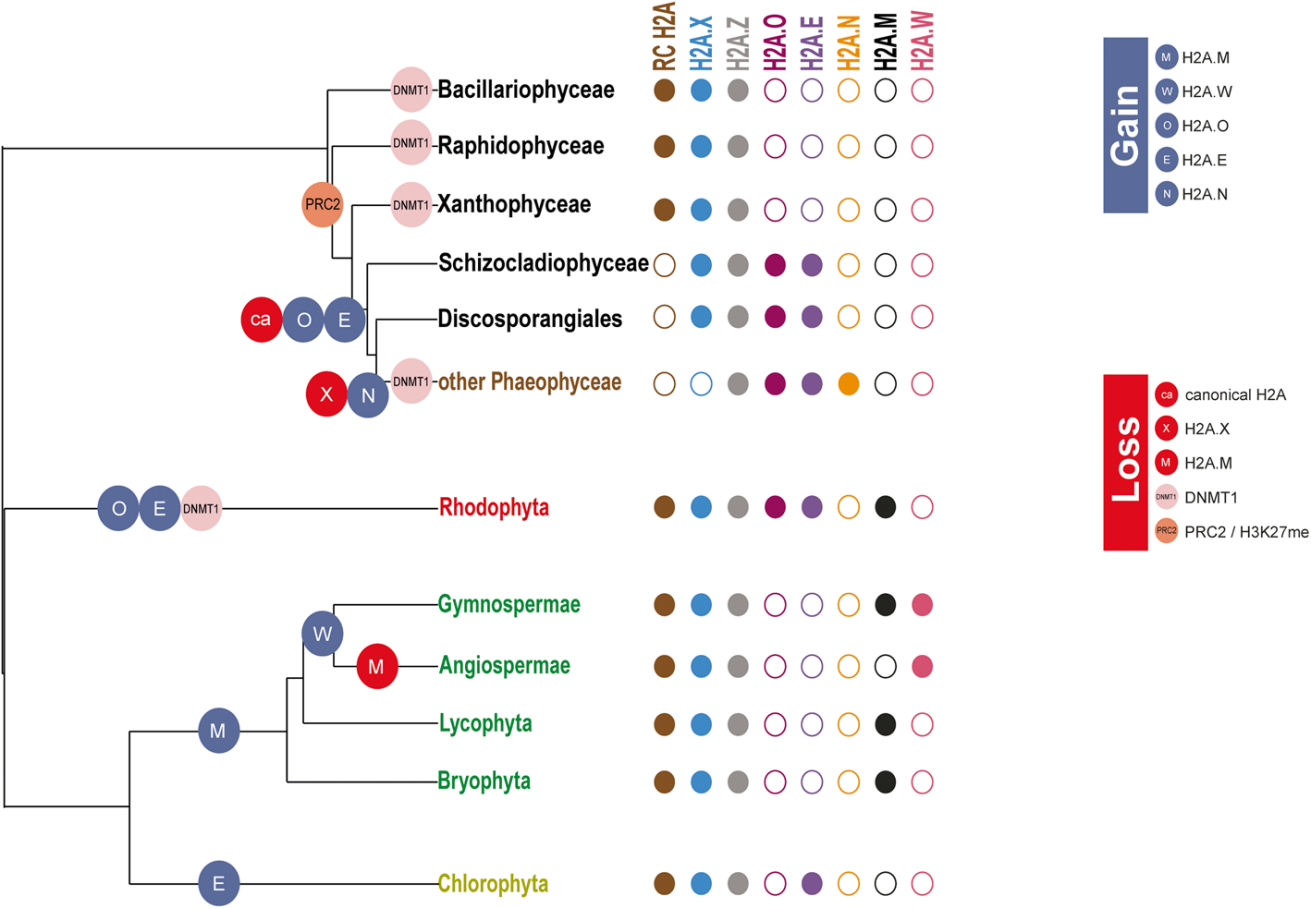
Evolution of some epigenetic features in photosynthetic organisms. The presented tree was constructed using TimeTree [44]. This figure displays the gain (dark blue circles on tree branches) or loss (red, orange or pink circles on tree branches) of some epigenetic features (H2A variants, PRC2 complex/H3K27 methylation, DNMT1) in photosynthetic organisms (algae and land plants). Representative species were used for each clade: *Heterosigma akashiwo* (Raphidophyceae); *Tribonema minus* (Xantophyceae and Chrysoparadoxophyceae); *Schizocladia ischiensis* (Schizocladiophyceae); *Choristocarpus tenellus* (Discoporangiales); *Ectocarpus* (other Phaeophycea); *Phaeodactylum tricornutum* (Diatoms); *Cyanidioschyzon merolae*, *Chondrus crispus* and *Porphyridium purpureum* (Rhodophyta); *Chlamydomonas reinhardtii, Ostreococcus lucimarinus* and *Ostreococcus tauri* (Chlorophyta); *Marchantia polymorpha* (Bryophyta), *Selaginella moellendorffii* (Lycophyta); *Picea abies* (Gymnospermae); *Arabidopsis thaliana* (Angiospermae). DNA methylation was not detected in *Ectocarpus* [39]. *Picea abies* has the DNMT1 homologue [45], DNA methylation [46] and the epigenetic mark H3K27me3 [47]. We performed a homology search for the catalytic subunit of the PRC2 complex, which did not retrieve any homologs in sister clades of Phaeophyceae. Methylation of H3K27 was not detected in *Ectocarpus*, consistently with the absence of homologs for PRC2 core proteins [14]. In vascular plants, *Selaginella moellendorffii* has a PRC2 complex [48] and a DNMT1 homolog [49] and DNA methylation has been reported in *Ceratopteris richardii*, another vascular plant [50]*.* For an easier display, we represented the gain of H2A.M based on the hypothesis that it evolved in land plants as H2A.W ancestor [9]. On the right side of the tree, the presence or absence of a H2A variant is depicted by a filled or unfilled circle, respectively. Each H2A variant is depicted in a different color with a code similar to that used in Fig. 1A

We investigated the tissue-specific expression of the various H2A variants throughout the complex life cycle of brown seaweeds as well as their regulation in response to environmental cues. The *H2A.Z, H2A.N* and *H2A.O* genes appeared to be ubiquitously expressed in most tissues and conditions for the analyzed species*.* In contrast, almost all *H2A.E* genes were highly expressed in sperm cells of *F. serratus* and *D. dichotoma* (Fig. 9A and Additional file 1: Fig. S9D). Female gametes (*i.e.* eggs) also expressed *H2A.E* genes but at a lower level, and not all of the genes were expressed (Fig. 9A and Additional file 1: Fig. S9D). To remodel chromatin during mammalian spermatogenesis, testis-specific histones are incorporated into the chromatin and become hyperacetylated before being replaced by protamines. They are then exchanged for oocyte-derived histones after fertilization (reviewed in [36]). In plants, male gametophyte development is associated with the incorporation of specific histone variants, such as the *Arabidopsis* sperm-specific variant H3.10 [27]. Moreover, pollen has specific histone post-translational modifications (reviewed in [37]). Furthermore, gametogenesis is associated with marked changes in genome expression and is therefore associated with chromatin modifications such as DNA methylation [38]. DNA methylation was not detected in *Ectocarpus* [39] but does occur in the diatom *P. tricornutum* [40] and at a low level in *Saccharina japonica* [41]*.* Diatoms and most brown seaweeds lack orthologues of MET1/DNMT1 (DNA METhyltransferase 1/DNA (cytosine-5)-MethylTransferase 1) [42, 43], the DNA methyltransferase involved in maintenance of CG methylation in plant and animal species. However, DNMT1 genes have been identified in *D. mesarthrocarpum* and two sister taxa of the brown algae *S. ischiensis* and *C. australica* [17]. Therefore, DNMT1 appears to have been lost after divergence of the Discosporangiales from other brown seaweeds and after the emergence of the H2A.E variant (Fig. 10). Given that brown seaweeds also lack gene-repression-associated histone PTMs such as H3K27me3 [14], they are likely to have developed alternative epigenetic mechanisms to compensate for the absence of these repressive marks. The expression pattern of H2A.E suggests that this variant might have acquired specific functions to enable epigenetic reprogramming during brown seaweed oogenesis and spermatogenesis. Epigenetic reprogramming is expected to occur in brown algae, probably through histone replacement and deposition of post-translational modifications. This study highlights the significance of understanding the impact of PTMs not only on RC histones but also on specific RI histone variants, which may fulfil unique functions.

## Conclusions

Brown seaweeds harbor a highly specific set of H2A variants, some of which are unique to this lineage and may compensate for the loss of some repressive epigenetic marks. The restricted distribution of these H2A variants among specific brown algal species suggests an adaptation to their various ecological niches and complex life cycles. In the future, it will be important to investigate the genomic localization of the H2A variants and, given the expanding applications of CRISPR-Cas9 mutagenesis [51], how loss of specific histone variants affects chromatin structure and gene expression.

## Methods

### Histone Identification in brown seaweed species using Phaeoexplorer genomic resources

Histone H2A protein sequence identification was performed by analysing:

* the 18 following brown seaweed species retrieved from the Phaeoexplorer website (https://phaeoexplorer.sb-roscoff.fr/home/): *Ascophyllum nodosum* (An), *Chordaria linearis* (Cl), *Choristocarpus tenellus* (Ct), *Desmarestia herbacea* (Dh), *Dictyota dichotoma* (Ddi), *Discosporangium mesarthrocarpum* (Dme), *Ectocarpus crouaniorum* (Ec), *Ectocarpus fasciculatus* (Ef), *Ectocarpus siliculosus* (Es), *Fucus serratus* (Fs), *Pleurocladia lacustris* (Pl), *Porterinema fluviatile* (Pf), *Pylaiella littoralis* (Pli), *Saccharina latissima* (Sl), *Sargassum fusiform* (Sf), *Scytosiphon promiscuus* (Sp), *Sphacelaria rigidula* (Sr) and *Undaria pinnatifida* (Up)
* and the four sister taxa species: *Chrysoparadoxa australica* (Ca), *Heterosigma akashiwo* (Ha)*, Schizocladia ischiensis* (Si) and *Tribonema minus* (Tm).

See Additional file 2: Table S9 for the publications corresponding to each genome.

Histone H2A homologs were identified in these 22 species with the BLASTp tool run on the protein databases (https://blast.sb-roscoff.fr/phaeoexplorer/) using the RC H2A protein sequence from the diatom *Phaeodactylum tricornutum*. To complete the analysis, genes and transcripts coding for the histones were retrieved from the genomes and predicted transcriptomes using BLAST (https://blast.sb-roscoff.fr/phaeoexplorer/) with default parameters (e-value 1e-05, matrix BLOSUM62, gap-open 11, gap-extend 1, filter F). Proteins encoded by the identified genes and transcripts were predicted with the Expasy web tool (https://web.expasy.org/translate/) and validated with PANTHER (https://www.pantherdb.org/), Interproscan [52] and Orthofinder [53]. All the identified protein sequences and corresponding transcripts IDs are reported in Additional file 2: Table S2. Information about genome quality for the 18 brown seaweed species and the four sister taxa species are detailed in Additional file 2: Table S9. Assembly quality for used genomes was sufficient to detect H2A variants comprehensively. Putative truncated proteins were identified based on protein alignments. We then manually curated genomes and transcriptomes to extend any truncated protein sequences and thereby correct any errors in the data due to incorrect gene models.

### Identification of histones in plants and animals

H2A protein sequences from *Homo sapiens* (Hs), *Mus musculus* (Mm), *Danio rerio* (Dr)*, Drosophila melanogaster* (Dm)*, Saccharomyces cerevisiae* (Sc), *Tetrahymena thermophila* (Tt) and *Zea mays* (Zm) correspond to protein sequences previously used in [54]. For *Chlamydomonas reinhardtii* (Cr)*, Chondrus crispus* (Crr), *Cyanidioschyzon merolae* (Cm), *Fragilariopsis cylindrus (*Fc), *Galdieria sulphuraria* (Gs), *Ostreococcus lucimarinus* (Ol), *Ostreococcus tauri* (Ot), *Phaeodactylum tricornutum* (Pt) and *Porphyridium purpureum *(Ppu), H2A protein sequences were retrieved from UniProt. For *Amborella trichopoda* (Atr)*, Arabidopsis thaliana* (At)*, Marchantia polymorpha* (Mp) and *Physcomitrella patens* (Ppa)*,* the H2A protein sequences were described in [9].

### Identification of histones in algae from the green and red algal clades

For *C. reinhardtii,* we used predicted proteins from [13] and [15]. Details regarding assignment of each protein to a variant type are provided in Additional file 3: Supplemental Methods. We identified H2A proteins with the BLAST tool run on the predicted protein database from the recently released dataset of deep genomics from multicellular red and green algae (BioProject: PRJNA924561) [24] using the H2A.Z protein sequence from *E. siliculosus*. We identified a H2A.E homologue in *A. amadelpha* with an incomplete sequence. However, genomic contigs for this species were not available (BioProject: PRJNA924561). We identified three H2A.E homologues in *C. fusiformis* and one H2A.O protein in *C. richterianum*. In this latter species, a tBLASTn search of genomic contigs did not identify any additional H2A proteins apart from the H2A.O variant.

### Phylogenetic analyses and protein sequence alignments

To generate the phylogenetic tree presented in Fig. 1B and in Additional file 1: Fig. S1B, selected sequences were first aligned in a multiple alignment with MUSCLE [55] using default parameters. IQ-TREE (http://iqtree.cibiv.univie.ac.at/) [56] was then applied with 1,000 bootstraps plus default parameters to generate the phylogenetic tree, which was drawn using the ITOL (Interactive Tree Of Life) tool [57]. The phylogenetic trees presented in Fig. 1B and Additional file 1: Fig. S1B were rooted with H2A.Z as an outgroup since it constitutes a distinct monophyletic clade among H2A variants [18] but is evolutionarily close to the other H2A variants. To compare the various H2A variants, multiple alignments were generated with Clustal Omega [58]. We used seven features that were based on motif analysis (αN helix, L1 loop, α2 helix, acidic patch and C-terminal ending) and domain length (L1 loop and docking domain) to classify H2A variants. Sequence characteristics for the different H2A variants are detailed in Additional file 3: Supplemental Methods.

### Analysis of shared synteny

Once genes coding for histone variants were identified, we analyzed the genetic neighborhood to identify putative orthologues in brown algal genomes. In *E. siliculosus,* we identified the four genes located on both sides of the *H2A.N* gene as well as the two genes upstream and five genes downstream of the *H2A.O* gene using the genome browser Jbrowse available on https://phaeoexplorer.sb-roscoff.fr/. For the 17 good quality genomes of Phaeophyceae species, we performed tBlastn searches using each flanking gene identified in *E. siliculosus.* This enabled us to identify orthologues and we confirmed their position using Jbrowse. The following species were excluded from this analysis due to a lower quality of the assembly for *H2A.N* and *H2A.O*-containing contigs: *A. nodosum, S. fusiform, S. rigidula, D. mesarthrocarpum, C. tenellus, T. minus* and *C. australica*.

### Analysis of consensus sequences

Either global or local multiple alignments generated with Clustal Omega [58] and consensus sequences were generated using Jalview Software [21]. Logos were generated with the WebLogo web server (https://weblogo.threeplusone.com/) [59] using the local multiple alignments of the sequences that were used to create the associated consensus. The amino acid height in the logo represents its relative frequency at its position.

### Identification of putative phosphorylation sites in H2A variants from *E. siliculosus*

Putative phosphorylation sites at serines, threonines and tyrosines residues of H2A variants (H2A.Z, H2A.N, H2A.O, H2A.E) from *E. siliculosus* were detected with NetPhos 3.1. Only scores higher than a specified threshold were retained as significant putative phosphorylation sites.

### Analysis of the expression for H2A variants

Differential expression data expressed in TPM (Transcripts Per Kilobase Million) were retrieved from RNA-Seq data generated for the following species: *F. serratus* (BioProject ID PRJNA731608, [60])*, P. lacustris* [17]*, P. fluviatile* [17], *D. herbacea* [17], *D. dichotoma* (Bioproject ID PRJNA356500) [61]*.* These data were obtained from the Phaeoexplorer project website (https://phaeoexplorer.sb-roscoff.fr/differential_expression/). For each species, all the RNA-seq data was mapped to the same reference genome and read counts generated using Salmon (version 1.3.0) [62]. For *D. dichotoma,* samples were female and male gametophytes, eggs collected 15 min after release, sperm cells 1 h after release, zygotes 1 h after release and embryos 8 h after fertilization [61] (Additional file 1: Fig. S9B)*.* For *F. serratus*, samples were female and male gametophyte thalli (referred to as female and male, respectively) as well as whole receptacles containing female eggs or male sperm [60] (Fig. 9A). For *Ectocarpus,* samples were collected from male (Ec457) and female (Ec460) *Ectocarpus* gametophyte lines and referred as female and male, respectively [63]. *D. herbacea, P. lacustris* and *P. fluviatile* samples were part of the project PRJEB72149 [17]. For *D. herbacea,* samples were female and male gametophytes grown in low light for one week before RNA extraction, cultures were transferred to high light to induce fertility [17]. For the fresh water species *P. lacustris* strain SAG 25.93 and *P. fluviatile* strain SAG 2381, samples correspond to whole thalli grown in Petri dish cultures prepared with either diluted (5%) natural seawater or undiluted natural seawater [17].

## Supplementary Information

Additional file 1: Fig S1-S9. Fig. S1: Phylogeny for species used in the present study and for H2A variants. Fig. S2: Characterization of RC H2A from various organisms and H2A.X from sister taxa of brown seaweeds. Fig. S3: Characterization of the H2A.N variant in brown seaweeds. Fig. S4: Characterization the H2A.Z proteins identified in brown seaweeds. Fig. S5: Characterization of the H2A.E proteins identified in brown seaweeds. Fig. S6: Characterization of the H2A.O proteins identified in brown seaweeds. Fig. S7: Analysis of the H2A variants in diatoms and in species from green and red algae. Fig. S8: Analysis of putative phosphorylations deposited on variants from Ectocarpus species 7. Fig. S9: Analysis of gene expression for the different H2A variants.

Additional file 2: Tables S1-S9. Table S1: List of the H2A proteins analyzed in the study. Table S2: List of the genes encoding each H2A variant. Table S3: Location of H2A genes on contigs of the studied species genomes. Table S4: Number of proteins for each H2A variant. Table S5: Analysis of motifs in the alpha N helix and the L1 loop for H2A.E and H2A.O variants. Table S6: Genes localised in the genomic vicinity of H2A.E coding genes. Table S7: Genes localised in the genomic vicinity of RC H2A- and H2A.X-coding genes in Heterosigma akashiwo. Table S8: Number of genes for each H2A variant. Table S9. List of brown seaweed species used for the project, the genome assemblies generated and analyzed.

Additional file 3: Supplemental Methods.

## Data Availability

Differential expression data were obtained from the Phaeoexplorer project website (https://phaeoexplorer.sb-roscoff.fr/differential_expression/). For F. serratus and D. dichotoma, samples correspond to the projects PRJNA731608 and PRJNA356500, respectively. The D. herbacea, P. lacustris and P. fluviatile samples were part of the project PRJEB72149. The deep genomic datasets from multicellular red and green algae were part of the project PRJNA924561.
