# Supplementary material for: Identification of novel H2A histone variants across diverse clades of algae
Source: Genome Biol. 2025 Sep 23;26:299. doi: 10.1186/s13059-025-03656-w (PMC12459048; doi:10.1186/s13059-025-03656-w)
Supplement: Supplementary file 1 — Additional file 1: Fig S1-S9. Fig. S1: Phylogeny for species used in the present study and for H2A variants. Fig. S2: Characterization of RC H2A from various organisms and H2A.X from sister taxa of brown seaweeds. Fig. S3: Characterization of the H2A.N variant in brown seaweeds. Fig. S4: Characterization the H2A.Z proteins identified in brown seaweeds. Fig. S5: Characterization of the H2A.E proteins identified in brown seaweeds. Fig. S6: Characterization of the H2A.O proteins identified in brown seaweeds. Fig. S7: Analysis of the H2A variants in diatoms and in species from green and red algae. Fig. S8: Analysis of putative phosphorylations deposited on variants from Ectocarpus species 7. Fig. S9: Analysis of gene expression for the different H2A variants. [file 13059_2025_3656_MOESM1_ESM.pdf]

A

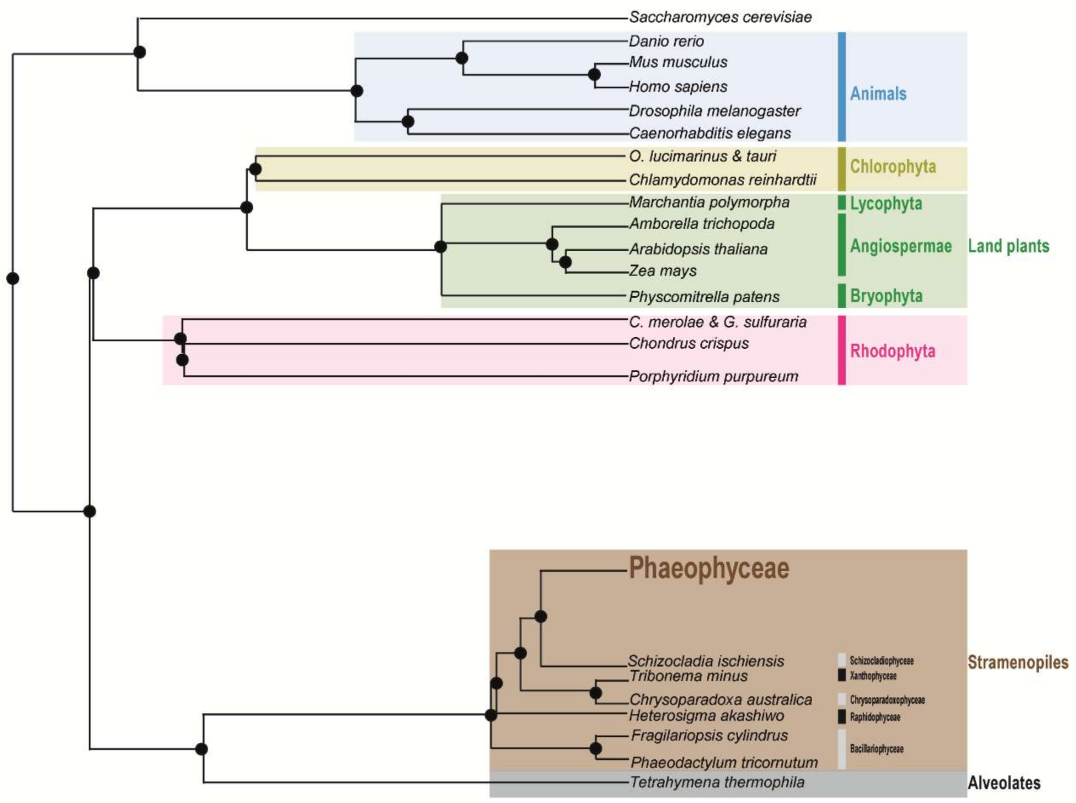

Saccharomyces cerevisiae
Danio rerio
Mus musculus
Homo sapiens
Drosophila melanogaster
Caenorhabditis elegans
Animals
O. lucimarinus & tauri
Chlamydomonas reinhardtii
Chlorophyta
Marchantia polymorpha
Lycophyta
Amborella trichopoda
Arabidopsis thaliana
Zea mays
Angiospermae
Land plants
Physcomitrella patens
Bryophyta
C. merolae & G. sulfuraria
Chondrus crispus
Porphyridium purpureum
Rhodophyta
Phaeophyceae
Schizocladia ischiensis
Schizocladiophyceae
Tribonema minus
Xanthophyceae
Stramenopiles
Chrysoparadoxa australica
Chrysoparadoxophyceae
Heterosigma akashiwo
Raphidophyceae
Fragilariopsis cylindrus
Phaeodactylum tricornutum
Bacillariophyceae
Tetrahymena thermophila
Alveolates

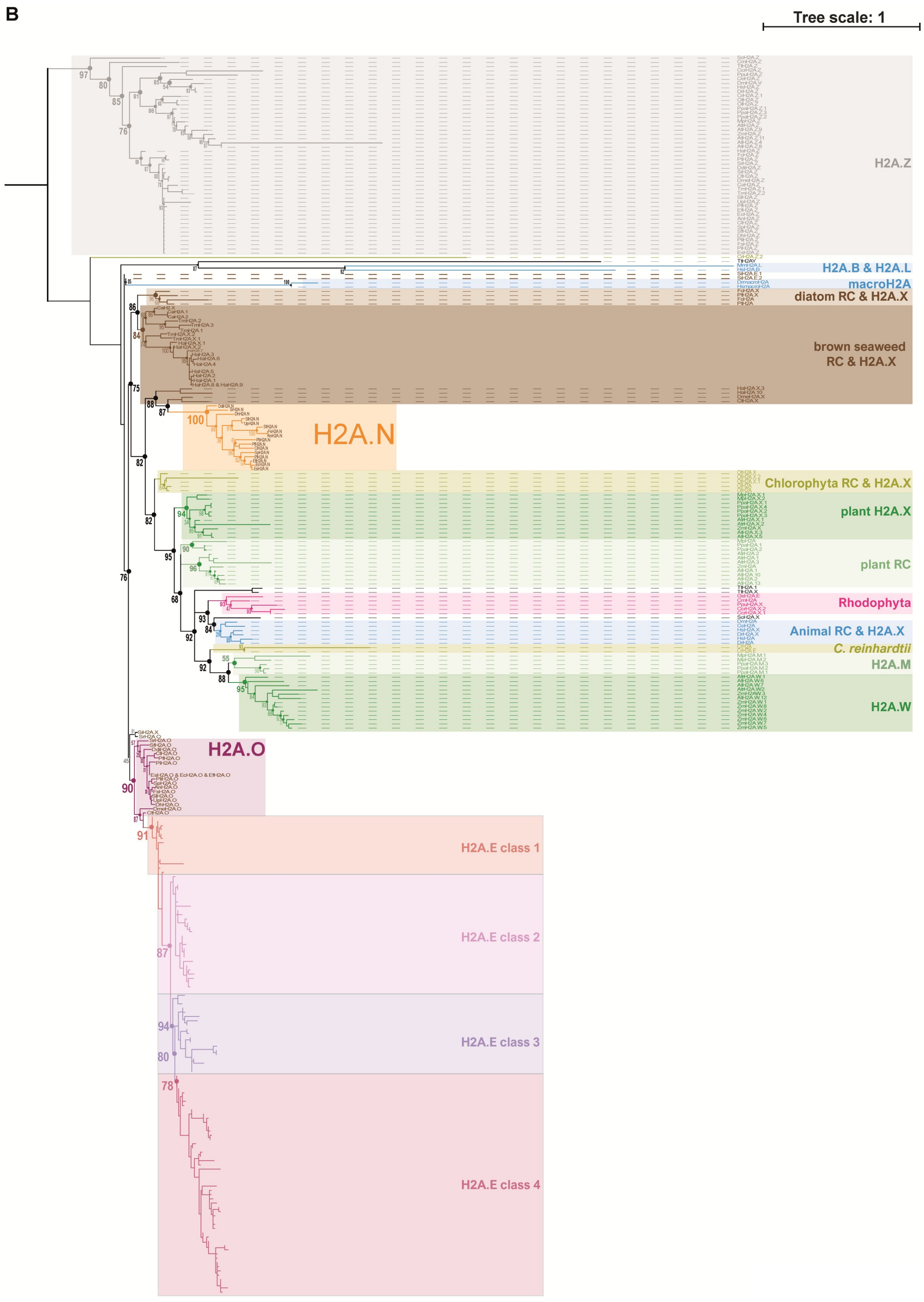

Fig. S1: Phylogeny for species used in the present study and for H2A variants.

(A) Schematic tree presenting the different representative species used to construct the phylogenetic tree for H2A variants presented in panel B. The presented tree was constructed using TimeTree [44]. For the Phaeophyceae, the detailed tree is presented in Fig. 1A. (*B*) Protein phylogeny based on maximum likelihood analysis for H2A variants from 18 brown seaweed species and four sister taxa along with representative species from yeast, Alveolates, animals, land plants, green and red algae. This phylogeny is represented as a phylogram with branch lengths scaled to divergence. The H2A.E proteins group into four main classes, classes 1 to 4. Bootstrap values are indicated for all nodes, with the exception of the H2A.E proteins. For a detailed phylogeny of the H2A.E variant, see Additional file 1: Fig. S5A-C. *Ascophyllum nodosum* (An), *Chordaria linearis* (Cl), *Choristocarpus tenellus* (Ct), *Chrysoparadoxa australica* (Ca), *Desmarestia herbacea* (Dh), *Dictyota dichotoma* (Ddi), *Discosporangium mesarthrocarpum* (Dme), *Ectocarpus crouaniorum* (Ec), *Ectocarpus fasciculatus* (Ef), *Ectocarpus siliculosus* (Es), *Fucus serratus* (Fse), *Heterosigma akashiwo* (Ha), *Pleurocladia lacustris* (Pla), *Porterinema fluviatile* (Pf), *Pylaiella littoralis* (Pli), *Saccharina latissima* (Sl), *Sargassum fusiform* (Sf), *Schizocladia ischiensis* (Si), *Scytosiphon promiscuus* (Sp), *Sphacelaria rigidula* (Sri), *Tribonema minus* (Tm) and *Undaria pinnatifida* (Up) for brown algae. *Amborella trichopoda* (Atr), *Arabidopsis thaliana* (At), *Chlamydomonas reinhardtii* (Cr), *Chondrus crispus* (Crr), *Cyanidioschyzon merolae* (Cm), *Danio rerio* (Dr), *Drosophila melanogaster* (Dm), *Fragilariopsis cylindrus* (Fc), *Galdieria sulphuraria* (Gs), *Homo sapiens* (Hs), *Marchantia polymorpha* (Mp), *Ostreococcus lucimarinus* (Ol), *Ostreococcus tauri* (Ot), *Phaeodactylum tricornutum* (Pt), *Physcomitrella patens* (Ppa), *Porphyridium purpureum* (Ppu), *Saccharomyces cerevisiae* (Sc), *Tetrahymena thermophila* (Tt) and *Zea mays* (Zm). Plant, animal, green and red algal species (A) and proteins (B) are displayed in dark green, blue, light green and pink respectively. For yeast and *T. thermophila*, they are displayed in black. The RC H2A and H2A.X proteins from Stramenopiles are displayed in brown. The H2A.Z proteins are displayed in grey.

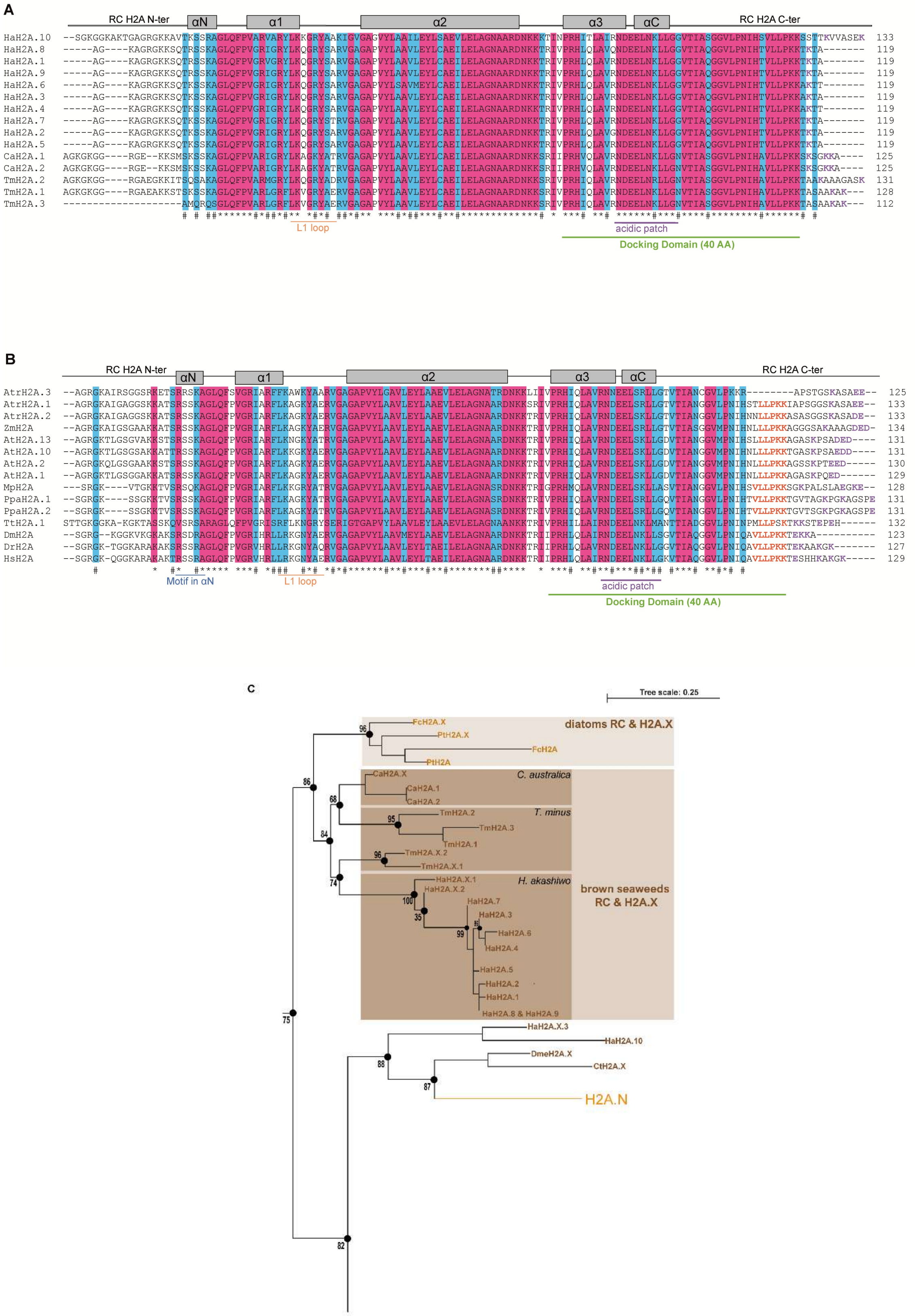
A
RC H2A N-ter
αN
α1
α2
α3
αC
RC H2A C-ter
HaH2A.10
HaH2A.8
HaH2A.1
HaH2A.9
HaH2A.6
HaH2A.3
HaH2A.4
HaH2A.7
HaH2A.2
HaH2A.5
CaH2A.1
CaH2A.2
TmH2A.2
TmH2A.1
TmH2A.3
L1 loop
acidic patch
Docking Domain (40 AA)
B
RC H2A N-ter
αN
α1
α2
α3
αC
RC H2A C-ter
AtrH2A.3
AtrH2A.1
AtrH2A.2
ZmH2A
AtH2A.13
AtH2A.10
AtH2A.2
AtH2A.1
MpH2A
PpaH2A.1
PpaH2A.2
TtH2A.1
DmH2A
DrH2A
HsH2A
Motif in αN
L1 loop
acidic patch
Docking Domain (40 AA)
C
Tree scale: 0.25
diatoms RC & H2A.X
FcH2A.X
PtH2A.X
FcH2A
PtH2A
C. australica
CaH2A.X
CaH2A.1
CaH2A.2
T. minus
TmH2A.2
TmH2A.3
TmH2A.1
TmH2A.X.2
TmH2A.X.1
H. akashiwo
HaH2A.X.1
HaH2A.X.2
HaH2A.7
HaH2A.3
HaH2A.6
HaH2A.4
HaH2A.5
HaH2A.2
HaH2A.1
HaH2A.8 & HaH2A.9
brown seaweeds RC & H2A.X
HaH2A.X.3
HaH2A.10
DmeH2A.X
CtH2A.X
H2A.N

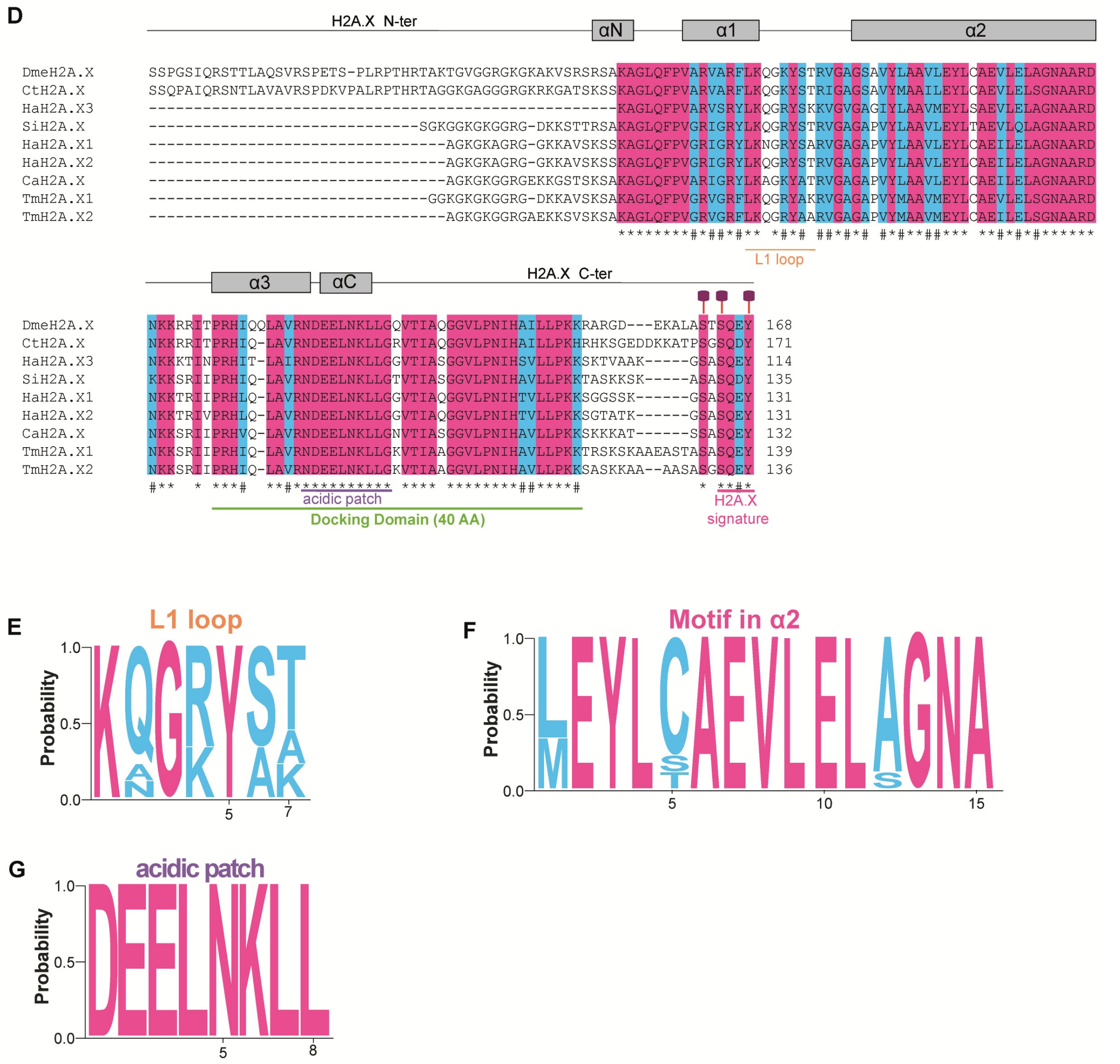

Fig. S2: Characterization of RC H2A from various organisms and H2A.X from sister taxa of brown seaweeds.

(A) Alignment of RC H2A proteins identified in *Heterosigma akashiwo* (Ha), *Tribonema minus* (Tm) and *Chrysoparadoxa australica* (Ca). These proteins have C-terminal tails enriched in lysines (in violet).

(B) Alignment of RC H2A proteins from representative animals and plants. These proteins have C-terminal tails enriched in lysine and acidic residues (K, D and E in violet) and a docking domain mostly ending with VLLPKK (in red). *Amborella trichopoda* (Atr), *Arabidopsis thaliana* (At), *Danio rerio* (Dr), *Drosophila melanogaster* (Dm, *Homo sapiens* (Hs), *Marchantia polymorpha* (Mp), *Physcomitrella patens* (Ppa), *Porphyridium purpureum* (Ppu), *Tetrahymena thermophila* (Tt) and *Zea mays* (Zm).

(C) Protein phylogeny based on maximum likelihood analysis for RC H2A and H2A.X proteins identified in diatoms and *C. australica, H. akashiwo* and *T. minus,* and *D. mesarthrocarpum* and *C. tenellus.* Bootstrap values obtained with IQ-TREE are shown. This tree focuses on the phylogeny of RC H2A and H2A.X proteins and was extracted from the tree presented in Additional file 1: Fig. Fig_S1B.

(D) Alignment of H2A.X proteins identified in the brown seaweeds *Discosporangium mesarthrocarpum* (Dme) and *Choristocarpus tenellus* (Ct) and in the sister taxa *Heterosigma akashiwo* (Ha)*, Tribonema minus* (Tm)*, Chrysoparadoxa australica* (Ca) and *Schizocladia ischiensis* (Si). Purple squares represent predicted phosphorylation sites.

For panels A, B and D, the L1 loop, acidic patch, docking domain and H2A.X signature are underlined in orange, purple, green and pink, respectively. The length of the docking domain is indicated (AA, amino acids). The helices are indicated by grey rectangles. Asterisks indicate a fully conserved residue and the hash conservation between residues with either strong or weak similar properties. N-ter, N-terminal tail; C-ter, C-terminal tail.

(E-G Logos of amino acid bias for the L1 loop (E), α2 helix (F) and acidic patch (G) for the H2A.X variant. Regions used to generate logos of amino acid bias are underlined in panel D.

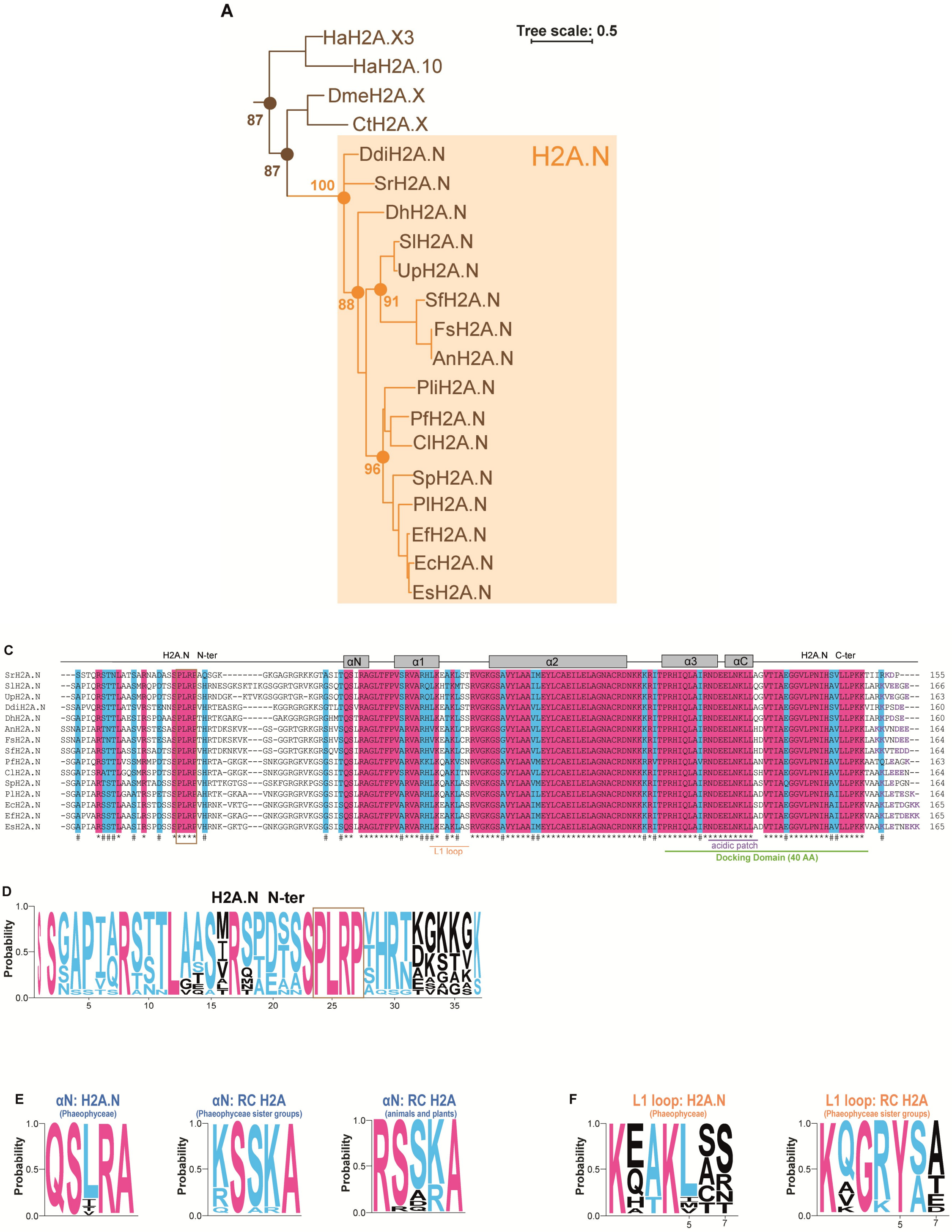
A
Tree scale: 0.5
HaH2A.X3
HaH2A.10
DmeH2A.X
CtH2A.X
H2A.N
DdiH2A.N
SrH2A.N
DhH2A.N
SlH2A.N
UpH2A.N
SfH2A.N
FsH2A.N
AnH2A.N
PliH2A.N
PfH2A.N
ClH2A.N
SpH2A.N
PlH2A.N
EfH2A.N
EcH2A.N
EsH2A.N
87
87
100
88
91
96
C
H2A.N N-ter
αN
α1
α2
α3
αC
H2A.N C-ter
L1 loop
acidic patch
Docking Domain (40 AA)
D
H2A.N N-ter
Probability
E
αN: H2A.N
(Phaeophyceae)
αN: RC H2A
(Phaeophyceae sister groups)
αN: RC H2A
(animals and plants)
F
L1 loop: H2A.N
(Phaeophyceae)
L1 loop: RC H2A
(Phaeophyceae sister groups)

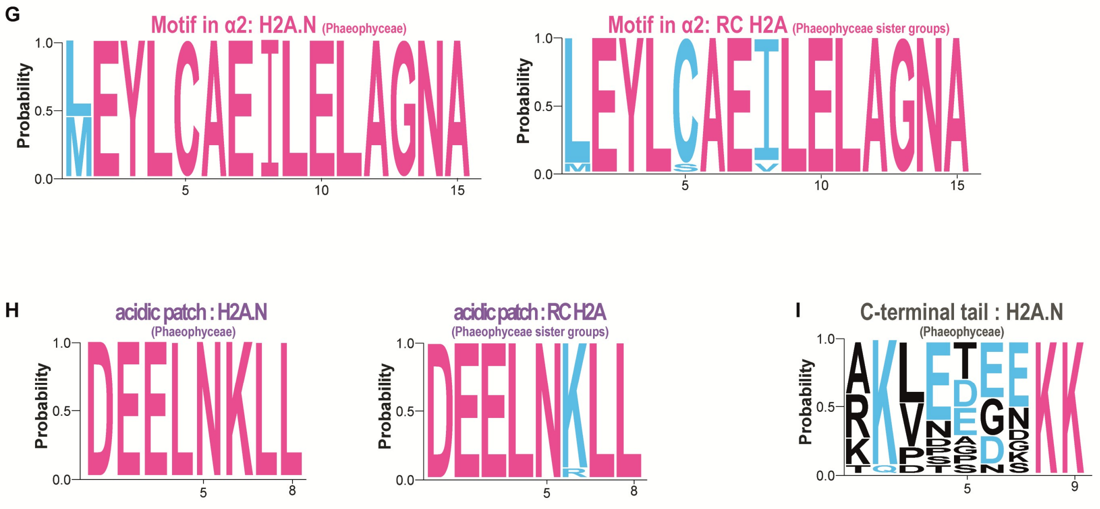

Fig. S3: Characterization of the H2A.N variant in brown seaweeds.

(A) Protein phylogeny based on maximum likelihood analysis for H2A.N proteins identified in brown seaweed species. Bootstrap values obtained with IQ-TREE are shown. This tree focuses on the phylogeny of H2A.N proteins and was extracted from the tree presented in Additional file 1: Fig. S1B.

(B) Alignment of H2A.N proteins identified in brown seaweeds and RC H2A proteins identified in *H. akashiwo, C. australica, T. minus*, animals and plants. The RC H2A from animals and plants have C-terminal tails with K, D and E amino acids (in violet). Most RC H2A proteins have a docking domain ending with VLLPKK (in red).

(*C*) Protein sequence alignment of brown algal H2A.N variants. The H2A.N variants have C-terminal tails enriched in K, D and E amino acids (in violet).

(D) Logo of amino acid bias for the long N-terminal tail of the H2A.N variant, which has a PLRP motif indicated in the protein alignment presented in panels B and C by a brown square.

(E) Logo of amino acid bias for the αN helix of the H2A.N proteins (left), RC H2A proteins from *H. akashiwo, C. australica, T. minus* (middle) and RC H2A proteins from representative animals and plants (right).

(F) Logo of amino acid bias for the L1 loop of the H2A.N proteins (left) and RC H2A proteins from *H. akashiwo, C. australica, T. minus* (right).

(G) Logo of amino acid bias for the α2 helix of the H2A.N proteins (left) and RC H2A proteins from *H. akashiwo, C. australica, T. minus* (right).

(H) Logo of amino acid bias for the acidic patch of the H2A.N proteins (left) and RC H2A proteins from *H. akashiwo, C. australica, T. minus* (right).

(I) Logo of amino acid bias for the C-terminal tail of the H2A.N proteins.

For panels E to I: Regions used to generate logos of amino acid bias for the αN helix (E), L1 loop (F), α2 helix (G), acid patch (H) and C-terminal tail (I) of H2A.N are indicated by blue, orange, pink, purple and grey squares, respectively in Fig. 3A. Regions used to generate logos of amino acid bias for the αN helix (E), L1 loop (F), α2 helix (G) and acid patch (H) of RC H2A proteins are indicated by blue, orange, pink and purple squares, respectively in Fig. 2A.

For the panels A, B, C and E: the L1 loop, acidic patch, and docking domain are underlined in orange, purple and green, respectively. The length of the docking domain is indicated (AA, amino acids). Helices are indicated by grey rectangles. Asterisks indicate a fully conserved residue and the hash conservation between residues with either strong or weak similar properties. N-ter, N-terminal tail; C-ter, C-terminal tail.

Ascophyllum nodosum (An), *Chordaria linearis* (Cl), *Choristocarpus tenellus* (Ct), *Chrysoparadoxa australica* (Ca), *Desmarestia herbacea* (Dh), *Dictyota dichotoma* (Ddi), *Discosporangium mesarthrocarpum* (Dme), *Ectocarpus crouaniorum* (Ec), *Ectocarpus fasciculatus* (Ef), *Ectocarpus siliculosus* (Es), *Fucus serratus* (Fse), *Heterosigma akashiwo* (Ha), *Pleurocladia lacustris* (Pla), *Porterinema fluviatile* (Pf), *Pylaiella littoralis* (Pli), *Saccharina latissima* (Sl), *Sargassum fusiform* (Sf), *Schizocladia ischiensis* (Si), *Scytosiphon promiscuus* (Sp), *Sphacelaria rigidula* (Sri), *Tribonema minus* (Tm) and *Undaria pinnatifida* (Up) for brown algae. *Amborella trichopoda* (Atr), *Arabidopsis thaliana* (At), *Danio rerio* (Dr), *Drosophila melanogaster* (Dm), *Homo sapiens* (Hs), *Marchantia polymorpha* (Mp), *Physcomitrella patens* (Ppa), *Saccharomyces cerevisiae* (Sc), *Tetrahymena thermophila* (Tt) and *Zea mays* (Zm).

A

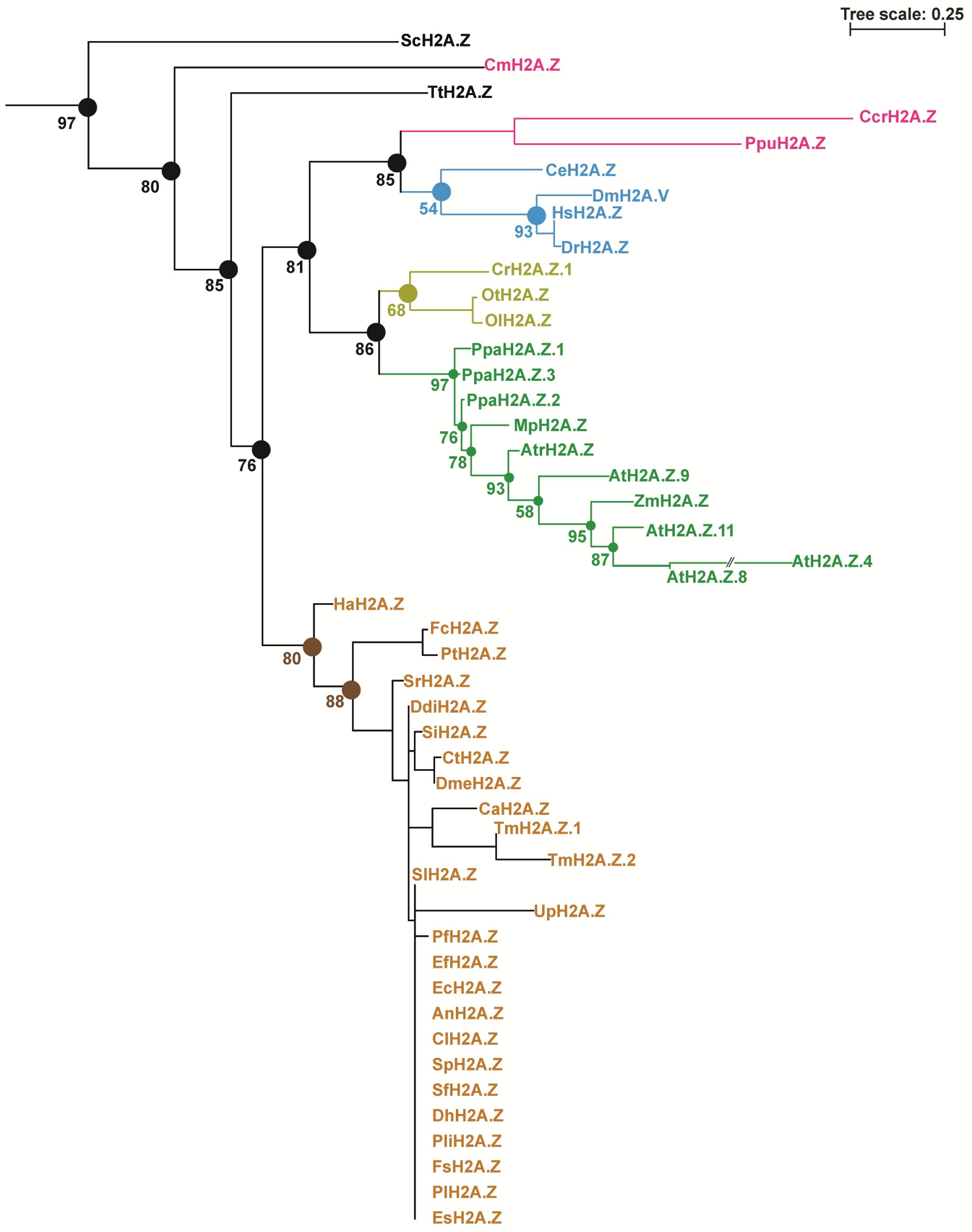
Tree scale: 0.25
ScH2A.Z
CmH2A.Z
TtH2A.Z
CcrH2A.Z
PpuH2A.Z
CeH2A.Z
DmH2A.V
HsH2A.Z
DrH2A.Z
CrH2A.Z.1
OtH2A.Z
OlH2A.Z
PpaH2A.Z.1
PpaH2A.Z.3
PpaH2A.Z.2
MpH2A.Z
AtrH2A.Z
AtH2A.Z.9
ZmH2A.Z
AtH2A.Z.11
AtH2A.Z.8
AtH2A.Z.4
HaH2A.Z
FcH2A.Z
PtH2A.Z
SrH2A.Z
DdiH2A.Z
SiH2A.Z
CtH2A.Z
DmeH2A.Z
CaH2A.Z
TmH2A.Z.1
TmH2A.Z.2
SlH2A.Z
UpH2A.Z
PfH2A.Z
EfH2A.Z
EcH2A.Z
AnH2A.Z
ClH2A.Z
SpH2A.Z
SfH2A.Z
DhH2A.Z
PliH2A.Z
FsH2A.Z
PlH2A.Z
EsH2A.Z
97
80
85
81
85
54
93
68
86
97
76
78
93
58
95
87
76
80
88

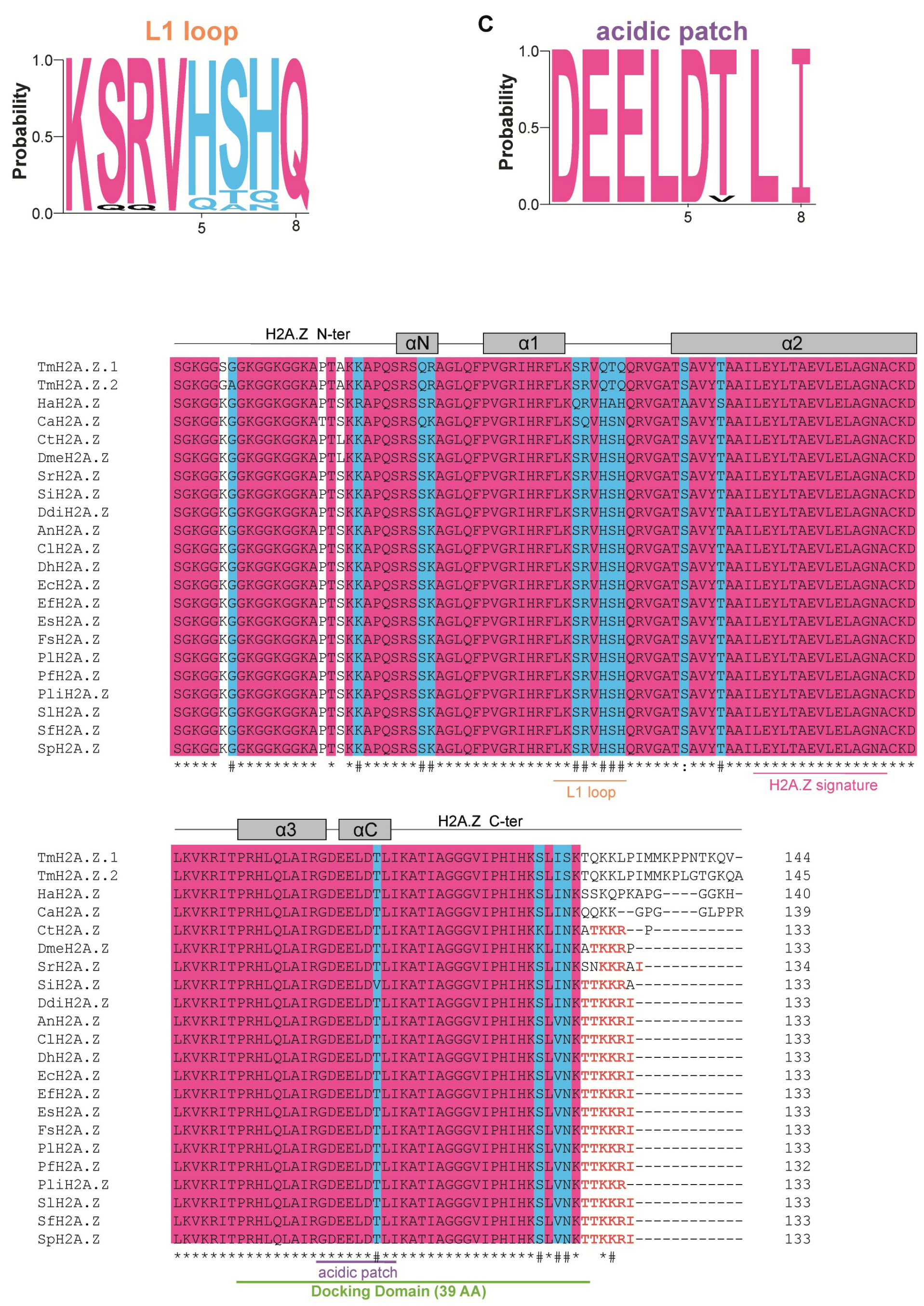

Fig. S4: Characterization the H2A.Z proteins identified in brown seaweeds.

(A) Protein phylogeny based on maximum likelihood analysis for H2A.Z proteins identified in brown seaweed species and representative species from animals, land plants or other algal species. Bootstrap values obtained with IQ-TREE are shown. This tree focuses on the phylogeny of H2A.Z proteins and was extracted from the tree presented in Additional file 1: Fig. S1B.

(B-C) Logo of amino acid bias for the L1 loop (B) and acidic patch (C) of the H2A.Z proteins (left) and RC H2A proteins from *H. akashiwo, C. australica, T. minus* (right). Regions used to generate

logos of amino acid bias for the L1 loop (B) and acid patch (C) of H2A.Z are indicated by orange and purple squares, respectively in Fig. 4A.

(D) Alignment of H2A.Z proteins identified in brown seaweeds and sister taxa species. The RC H2A from animals and plants have C-terminal tails with K, D and E amino acids (in violet). Most H2A.Z proteins have a C-terminal tail ending with TTKKRI (in red). The L1 loop, acidic patch, and docking domain are underlined in orange, purple and green, respectively. The H2A.Z signature in the α2 helix is underlined in pink. The length of the docking domain is indicated (AA, amino acids). Helices are indicated by grey rectangles. Asterisks indicate a fully conserved residue and the hash conservation between residues with either strong or weak similar properties. N-ter, N-terminal tail; C-ter, C-terminal tail.

Ascophyllum nodosum (An), *Chordaria linearis* (Cl), *Choristocarpus tenellus* (Ct), *Chrysoparadoxa australica* (Ca), *Desmarestia herbacea* (Dh), *Dictyota dichotoma* (Ddi), *Discosporangium mesarthrocarpum* (Dme), *Ectocarpus crouaniorum* (Ec), *Ectocarpus fasciculatus* (Ef), *Ectocarpus siliculosus* (Es), *Fucus serratus* (Fse), *Heterosigma akashiwo* (Ha), *Pleurocladia lacustris* (Pla), *Porterinema fluviatile* (Pf), *Pylaiella littoralis* (Pli), *Saccharina latissima* (Sl), *Sargassum fusiform* (Sf), *Schizocladia ischiensis* (Si), *Scytosiphon promiscuus* (Sp), *Sphacelaria rigidula* (Sri), *Tribonema minus* (Tm) and *Undaria pinnatifida* (Up) for brown algae. *Amborella trichopoda* (Atr), *Arabidopsis thaliana* (At), *Danio rerio* (Dr), *Drosophila melanogaster* (Dm), *Homo sapiens* (Hs), *Marchantia polymorpha* (Mp), *Physcomitrella patens* (Ppa), *Saccharomyces cerevisiae* (Sc), *Tetrahymena thermophila* (Tt) and *Zea mays* (Zm).

A

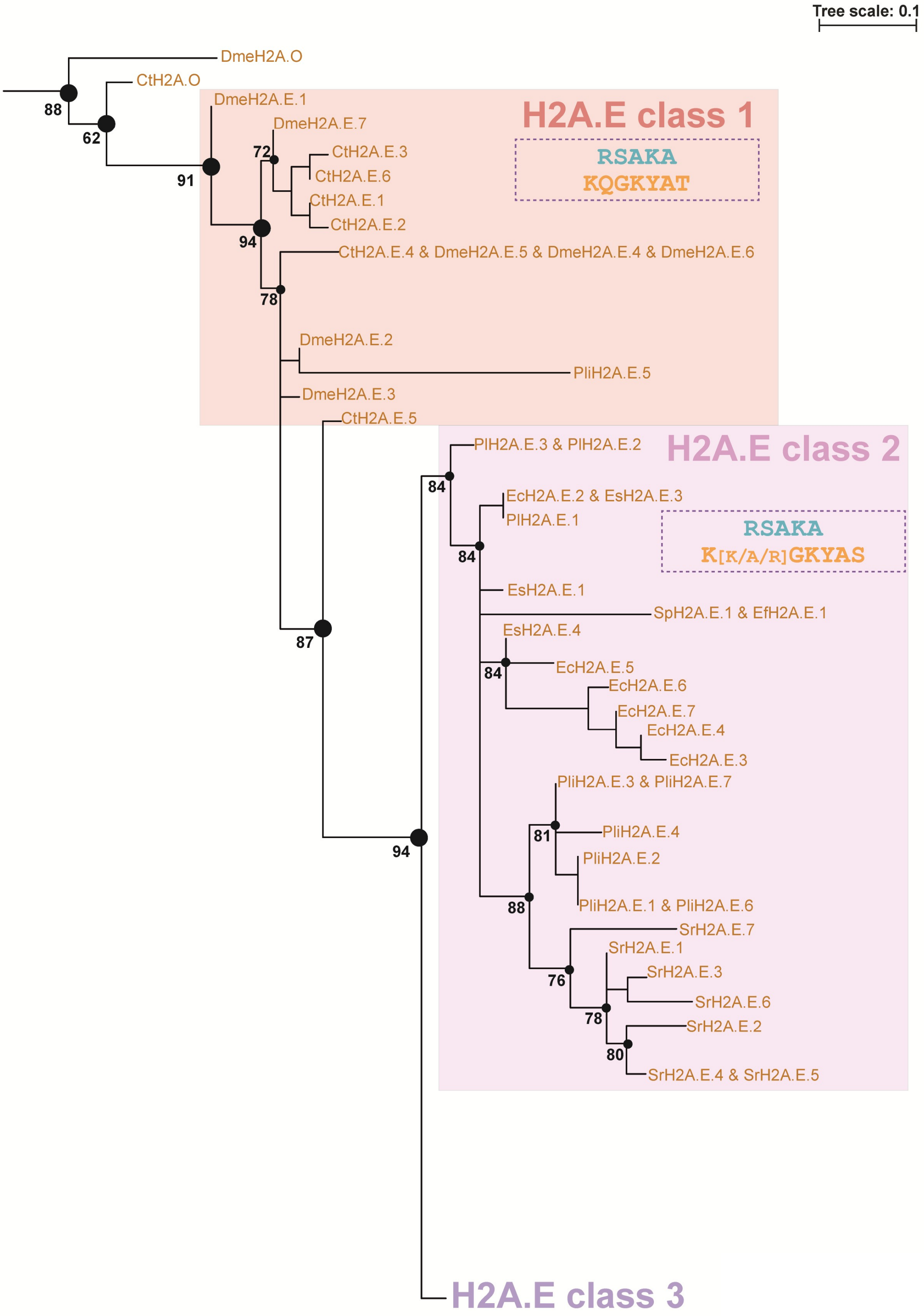

Tree scale: 0.1
DmeH2A.O
CtH2A.O
88
62
91
DmeH2A.E.1
H2A.E class 1
RSAKA
KQGKYAT
DmeH2A.E.7
72
CtH2A.E.3
CtH2A.E.6
CtH2A.E.1
CtH2A.E.2
94
CtH2A.E.4 & DmeH2A.E.5 & DmeH2A.E.4 & DmeH2A.E.6
78
DmeH2A.E.2
PliH2A.E.5
DmeH2A.E.3
CtH2A.E.5
PlH2A.E.3 & PlH2A.E.2
H2A.E class 2
84
EcH2A.E.2 & EsH2A.E.3
PlH2A.E.1
RSAKA
K[K/A/R]GKYAS
84
EsH2A.E.1
SpH2A.E.1 & EfH2A.E.1
87
EsH2A.E.4
EcH2A.E.5
84
EcH2A.E.6
EcH2A.E.7
EcH2A.E.4
EcH2A.E.3
PliH2A.E.3 & PliH2A.E.7
81
PliH2A.E.4
94
PliH2A.E.2
PliH2A.E.1 & PliH2A.E.6
88
SrH2A.E.7
SrH2A.E.1
76
SrH2A.E.3
SrH2A.E.6
78
SrH2A.E.2
80
SrH2A.E.4 & SrH2A.E.5
H2A.E class 3

B

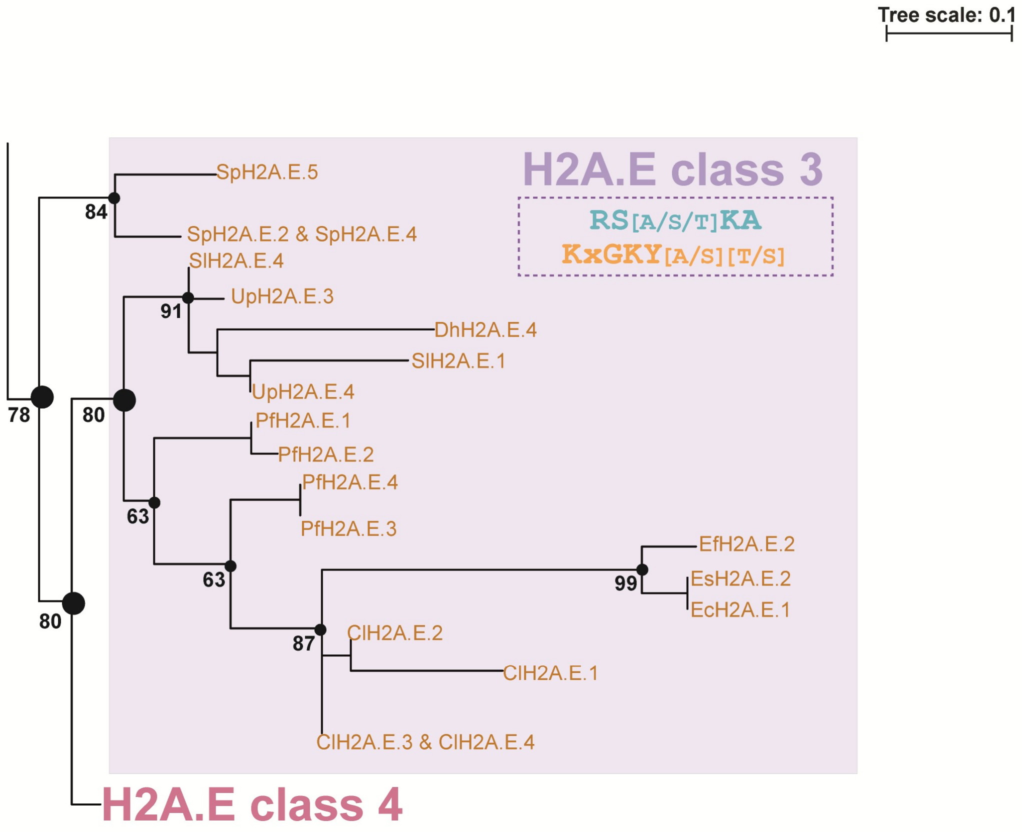

Tree scale: 0.1
H2A.E class 3
RS[A/S/T]KA
KxGKY[A/S][T/S]
SpH2A.E.5
84
SpH2A.E.2 & SpH2A.E.4
SlH2A.E.4
UpH2A.E.3
91
DhH2A.E.4
SlH2A.E.1
UpH2A.E.4
78
80
PfH2A.E.1
PfH2A.E.2
PfH2A.E.4
63
PfH2A.E.3
63
EfH2A.E.2
99
EsH2A.E.2
EcH2A.E.1
80
ClH2A.E.2
87
ClH2A.E.1
ClH2A.E.3 & ClH2A.E.4
H2A.E class 4

C

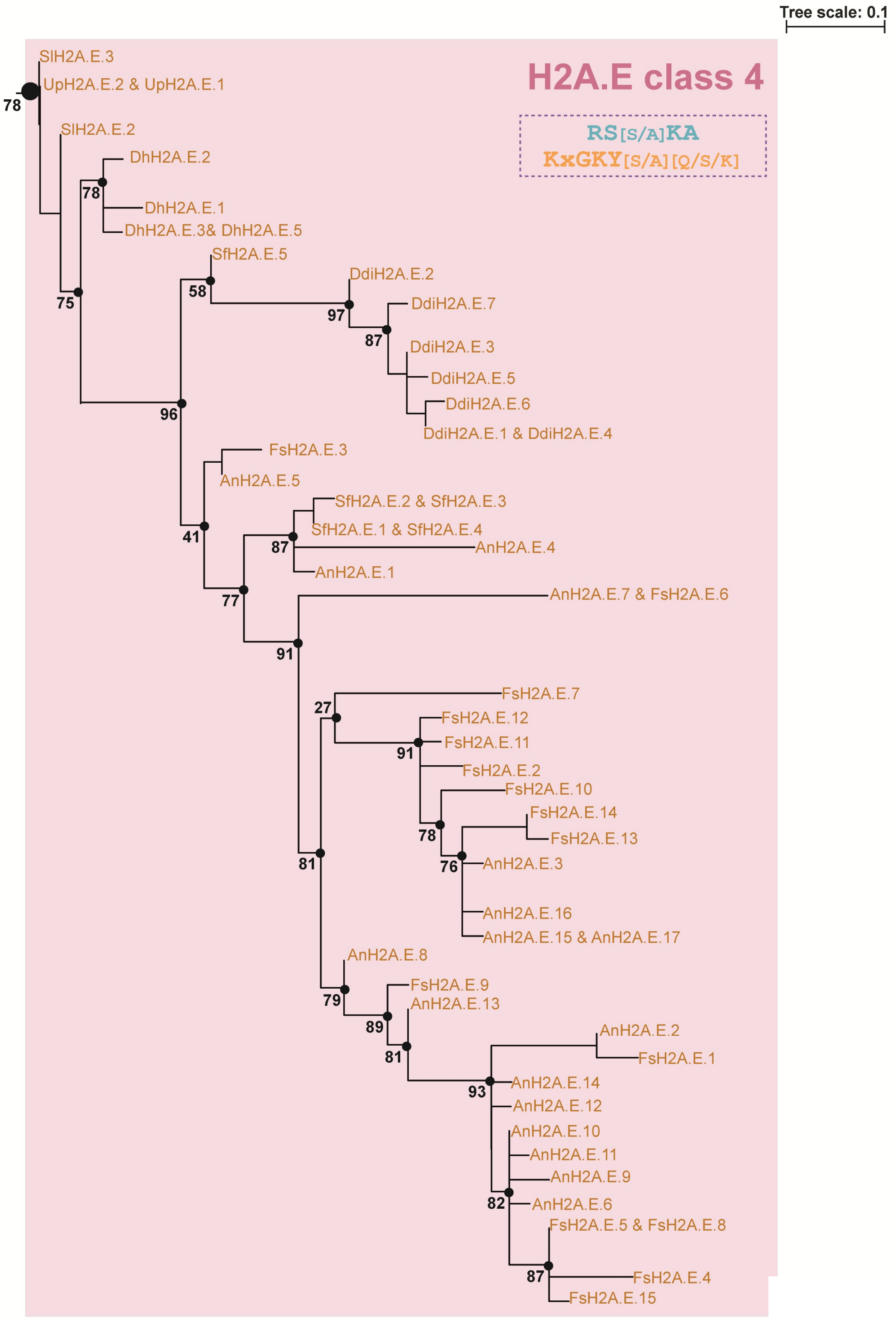
Tree scale: 0.1
H2A.E class 4
RS[S/A]KA
KxGKY[S/A][Q/S/K]
SlH2A.E.3
UpH2A.E.2 & UpH2A.E.1
SlH2A.E.2
DhH2A.E.2
DhH2A.E.1
DhH2A.E.3& DhH2A.E.5
SfH2A.E.5
DdiH2A.E.2
DdiH2A.E.7
DdiH2A.E.3
DdiH2A.E.5
DdiH2A.E.6
DdiH2A.E.1 & DdiH2A.E.4
FsH2A.E.3
AnH2A.E.5
SfH2A.E.2 & SfH2A.E.3
SfH2A.E.1 & SfH2A.E.4
AnH2A.E.4
AnH2A.E.1
AnH2A.E.7 & FsH2A.E.6
FsH2A.E.7
FsH2A.E.12
FsH2A.E.11
FsH2A.E.2
FsH2A.E.10
FsH2A.E.14
FsH2A.E.13
AnH2A.E.3
AnH2A.E.16
AnH2A.E.15 & AnH2A.E.17
AnH2A.E.8
FsH2A.E.9
AnH2A.E.13
AnH2A.E.2
FsH2A.E.1
AnH2A.E.14
AnH2A.E.12
AnH2A.E.10
AnH2A.E.11
AnH2A.E.9
AnH2A.E.6
FsH2A.E.5 & FsH2A.E.8
FsH2A.E.4
FsH2A.E.15
78
78
75
58
97
87
96
41
87
77
91
27
91
78
76
81
79
89
81
93
82
87

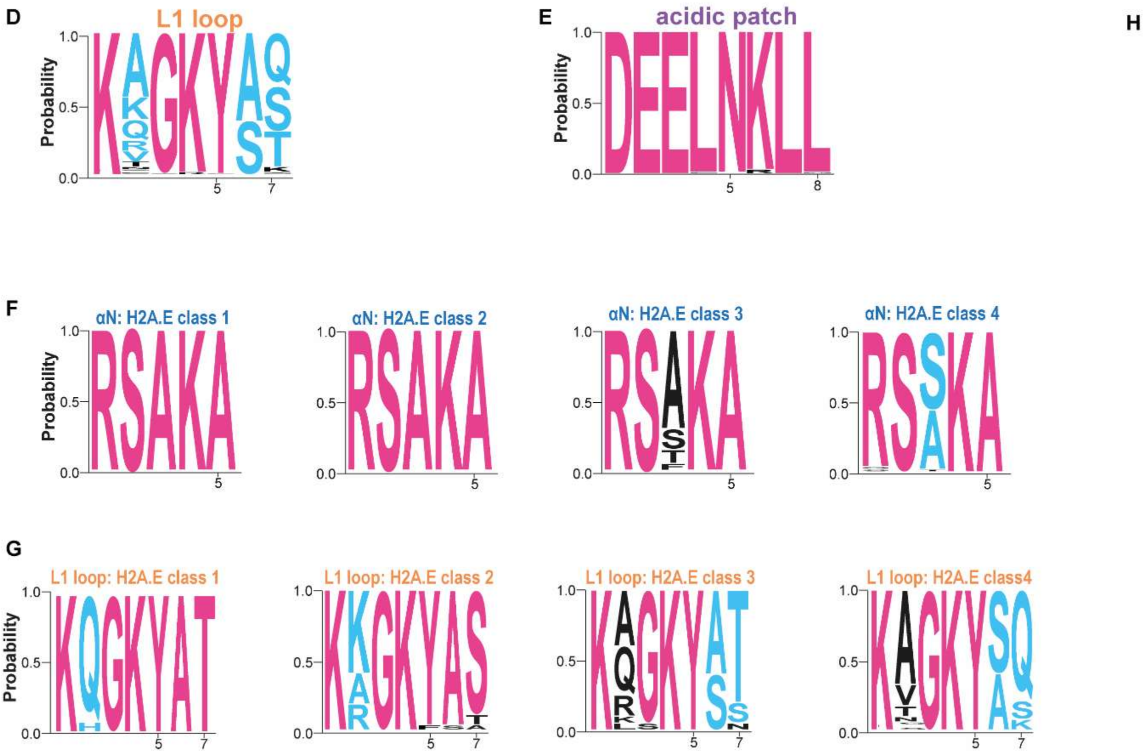

D
L1 loop
Probability
E
acidic patch
H
F
αN: H2A.E class 1
αN: H2A.E class 2
αN: H2A.E class 3
αN: H2A.E class 4
G
L1 loop: H2A.E class 1
L1 loop: H2A.E class 2
L1 loop: H2A.E class 3
L1 loop: H2A.E class4

H

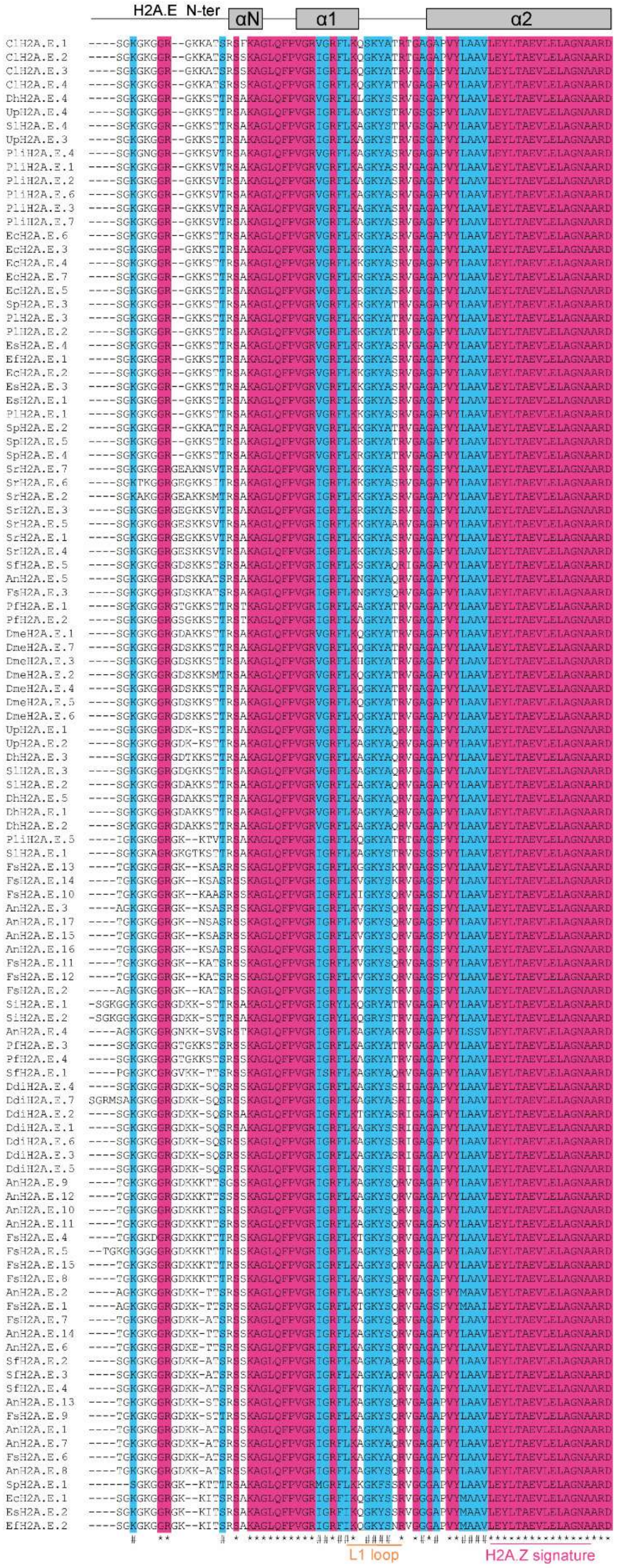

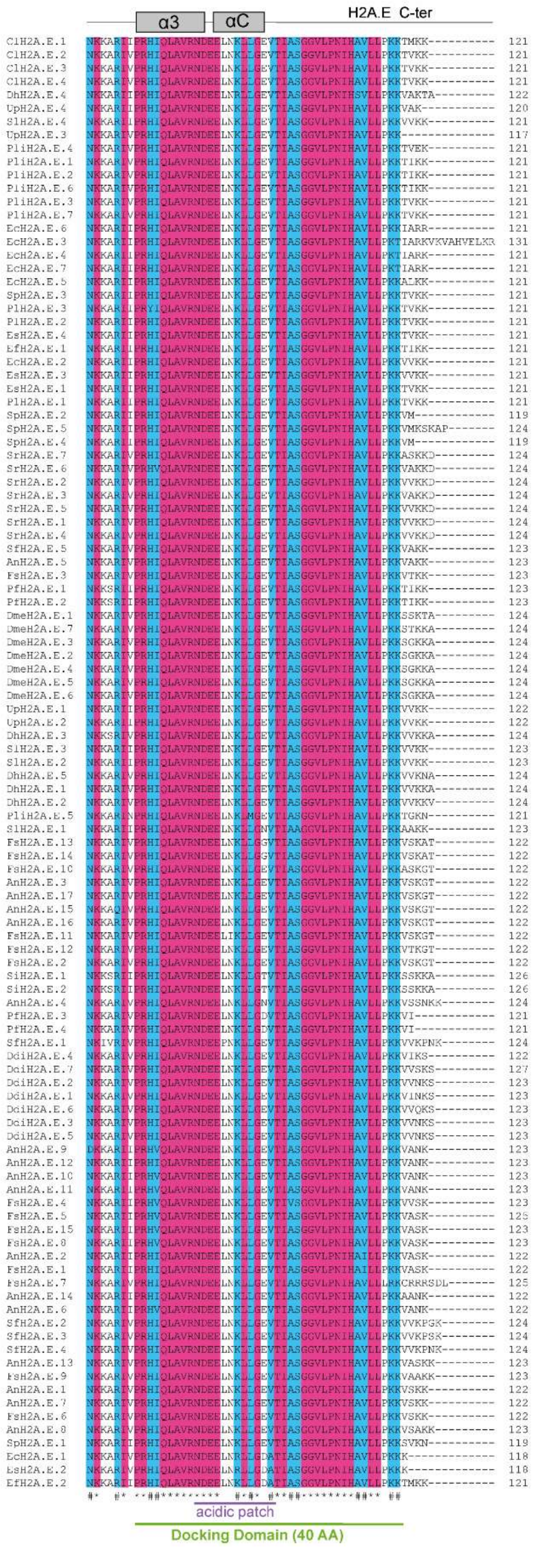

Fig. S5: Characterization of the H2A.E proteins identified in brown seaweeds.

(A-C) Protein phylogeny based on maximum likelihood analysis for H2A.E proteins of classes 1 and 2 (A), class 3 (B) and class 4 (C) identified in brown seaweed species. Bootstrap values obtained with IQ-TREE are shown. This tree focuses on the phylogeny of H2A.E proteins and was

extracted from the tree presented in Additional file 1: Fig. S1B. To emphasize differences in proteins from various classes of variants, the consensus sequence is indicated in blue for the αN helix and in orange for the L1 loop.

(D-E) Logos of amino acid bias for the L1 loop (D) and acidic patch (E) of H2A.E proteins from classes 1, 2, 3 and 4. Regions used to generate logos of amino acid bias for the L1 loop (D) and acid patch (E) of H2A.E are indicated by orange and purple squares, respectively in Fig. 5A.

(F-G) Logos of amino acid bias for the αN helix (F) and the L1 loop (G) of H2A.E proteins from classes 1 and 2 and 3 and 4.

(H) Alignment of H2A.E proteins. The L1 loop, acidic patch, docking domain and H2A.Z signature in the α2 helix are underlined in orange, purple, green and pink, respectively. The length of the docking domain is indicated (AA, amino acids). Helices are indicated by grey rectangles. Asterisks indicate a fully conserved residue and the hash conservation between residues with either strong or weak similar properties. N-ter, N-terminal tail; C-ter, C-terminal tail. *Ascophyllum nodosum* (An), *Chordaria linearis* (Cl), *Choristocarpus tenellus* (Ct), *Desmarestia herbacea* (Dh), *Dictyota dichotoma* (Ddi), *Discosporangium mesarthrocarpum* (Dme), *Ectocarpus crouaniorum* (Ec), *Ectocarpus fasciculatus* (Ef), *Ectocarpus siliculosus* (Es), *Fucus serratus* (Fse), *Pleurocladia lacustris* (Pla), *Porterinema fluviatile* (Pf), *Pylaiella littoralis* (Pli), *Saccharina latissima* (Sl), *Sargassum fusiform* (Sf), *Schizocladia ischiensis* (Si), *Scytosiphon promiscuus* (Sp), *Sphacelaria rigidula* (Sri) and *Undaria pinnatifida* (Up).

A

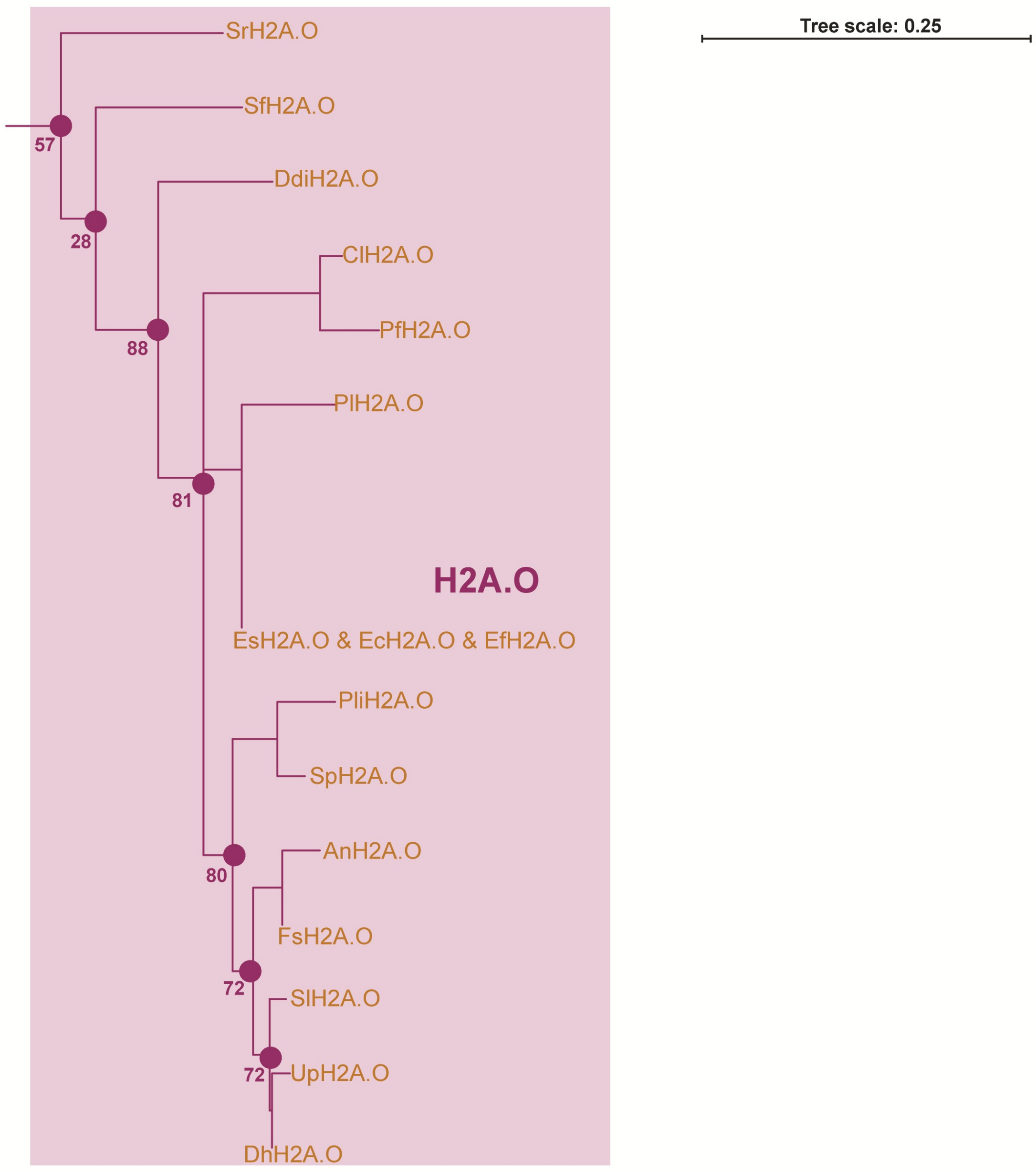
SrH2A.O
SfH2A.O
DdiH2A.O
ClH2A.O
PfH2A.O
PlH2A.O
H2A.O
EsH2A.O & EcH2A.O & EfH2A.O
PliH2A.O
SpH2A.O
AnH2A.O
FsH2A.O
SlH2A.O
UpH2A.O
DhH2A.O
57
28
88
81
80
72
72
Tree scale: 0.25

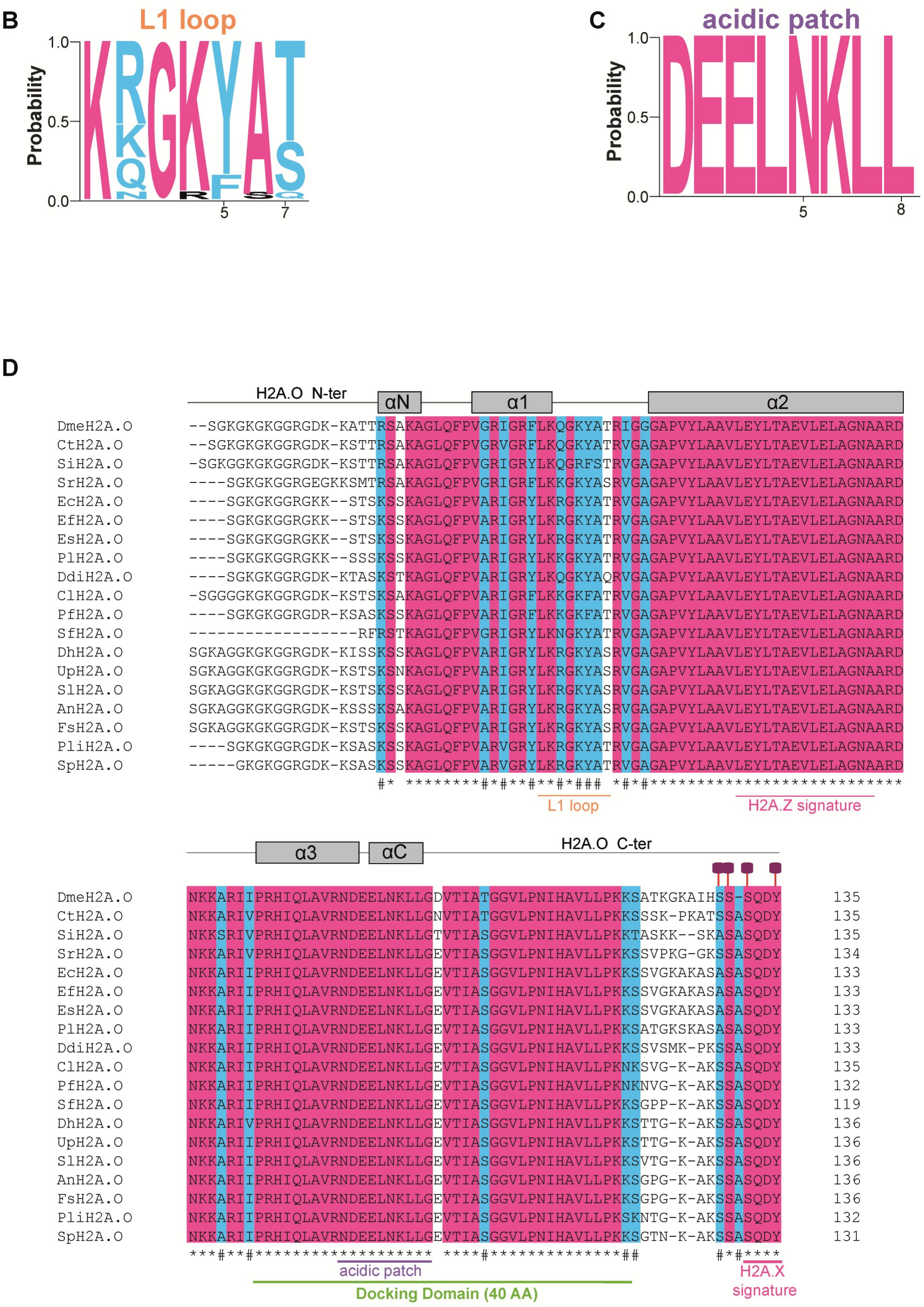

Fig. S6: Characterization of the H2A.O proteins identified in brown seaweeds.

(A) Protein phylogeny based on maximum likelihood analysis for H2A.O proteins identified in brown seaweed species. Bootstrap values obtained with IQ-TREE are shown. This tree focuses on the phylogeny of H2A.O proteins and was extracted from the tree presented in Additional file 1: Fig. S1B.

(B-C) Logos of amino acid bias for the L1 loop (B) and acidic patch (C) of H2A.O proteins from brown seaweeds. Regions used to generate logos of amino acid bias for the L1 loop (B) and acid patch (C) of H2A.O are indicated by orange and purple squares, respectively in Fig. 6A.

(D) Alignment of H2A.O proteins. The L1 loop, acidic patch, docking domain and H2A.Z signature in the α2 helix are underlined in orange, purple, green and pink, respectively. The length of the docking domain is indicated (AA, amino acids). Helices are indicated by grey rectangles. Asterisks indicate a fully conserved residue and the hash conservation between residues with either strong or weak similar properties. N-ter, N-terminal tail; C-ter, C-terminal tail. *Ascophyllum nodosum* (An), *Chordaria linearis* (Cl), *Choristocarpus tenellus* (Ct), *Desmarestia herbacea* (Dh), *Dictyota dichotoma* (Ddi), *Discosporangium mesarthrocarpum* (Dme), *Ectocarpus crouaniorum* (Ec), *Ectocarpus fasciculatus* (Ef), *Ectocarpus siliculosus* (Es), *Fucus serratus* (Fse), *Pleurocladia lacustris* (Pla), *Porterinema fluviatile* (Pf), *Pylaiella littoralis* (Pli), *Saccharina latissima* (Sl), *Sargassum fusiform* (Sf), *Schizocladia ischiensis* (Si), *Scytosiphon promiscuus* (Sp), *Sphacelaria rigidula* (Sri) and *Undaria pinnatifida* (Up).

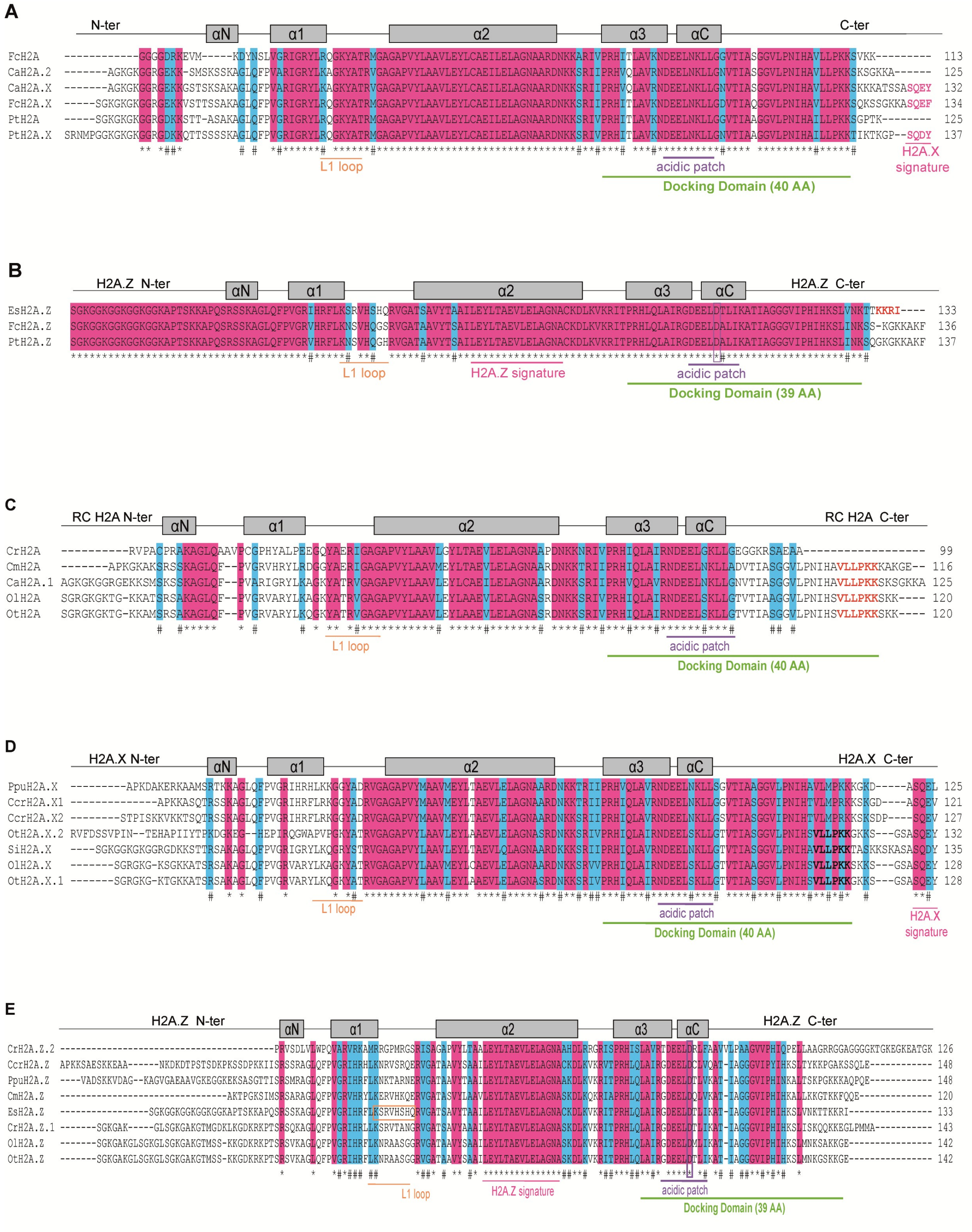
A
N-ter
αN
α1
α2
α3
αC
C-ter
L1 loop
acidic patch
Docking Domain (40 AA)
H2A.X signature
B
H2A.Z N-ter
H2A.Z C-ter
L1 loop
H2A.Z signature
acidic patch
Docking Domain (39 AA)
C
RC H2A N-ter
RC H2A C-ter
L1 loop
acidic patch
Docking Domain (40 AA)
D
H2A.X N-ter
H2A.X C-ter
L1 loop
acidic patch
Docking Domain (40 AA)
H2A.X signature
E
H2A.Z N-ter
H2A.Z C-ter
L1 loop
H2A.Z signature
acidic patch
Docking Domain (39 AA)

F

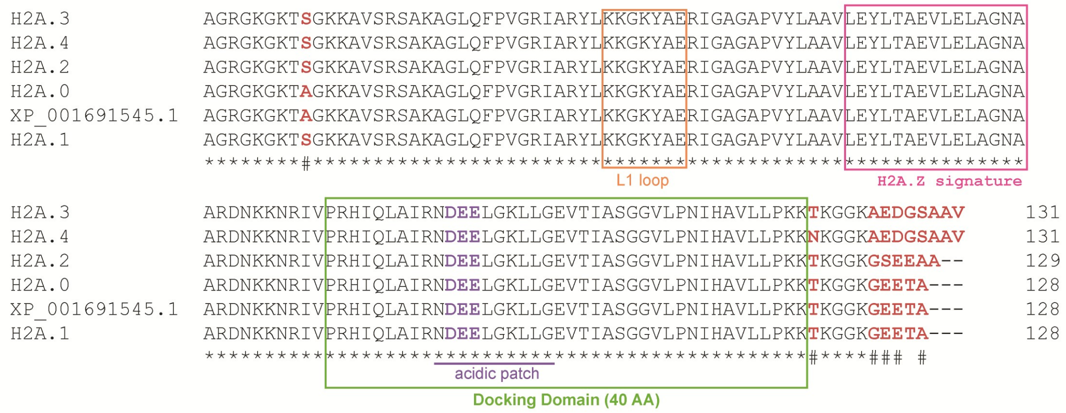

Fig. S7: Analysis of the H2A variants in diatoms and in species from green and red algae.
(A-B) Alignment of the H2A.X and RC H2A variants (A), the H2A.Z variant (B) from two diatom species.
(C-E) Alignment of RC H2A (C), H2A.X (D) and H2A.Z (E) proteins from the brown seaweed *E. siliculosus,* the sister taxa species *S. ischiensis* and *C. australica* as well as from species of green algae (*C. reinhardtii, O. lucimarinus*, *O. tauri*) and red algae (*P. purpureum, C. merolae, C. fusiformis, C. crispus)*. The L1 loop, acidic patch, docking domain and the H2A.X signature and the H2A.Z signature in the α2 helix are underlined in orange, purple, green and pink, respectively. The length of the docking domain is indicated (AA, amino acids). The helices are indicated by grey rectangles. Asterisks indicate a fully conserved residue and the hash conservation between residues with either strong or weak similar properties. N-ter, N-terminal tail; C-ter, C-terminal tail. *Fragilariopsis cylindrus (*Fc), *Phaeodactylum tricornutum* (Pt), *Chrysoparadoxa australica* (Ca), *Chlamydomonas reinhardtii* (Cr)*, Chondrus crispus* (Crr), *Cyanidioschyzon merolae* (Cm), *Ectocarpus siliculosus* (Es), *Ostreococcus lucimarinus* (Ol), *Ostreococcus tauri* (Ot), *Porphyridium purpureum* (Ppu), *Schizocladia ischiensis* (Si), *Catenella fusiformis* (Cf).
(F) Protein sequence alignment of the various published sequences for *Chlamydomonas reinhardtii.* The L1 loop, H2A.Z signature in the α2 helix and docking domain are indicated by a rectangle in orange, green and pink respectively; the acidic patch is underlined in purple. The length of the docking domain is indicated (AA, amino acids). Asterisks indicate a fully conserved residue and the hash conservation between residues with either strong or weak similar properties. The five proteins (H2A.0, H2A.1, H2A.2, H2A.3, H2A.4) predicted by [15] displayed minor sequence variations and are highly similar to XP_001691545.1 from Uniprot.

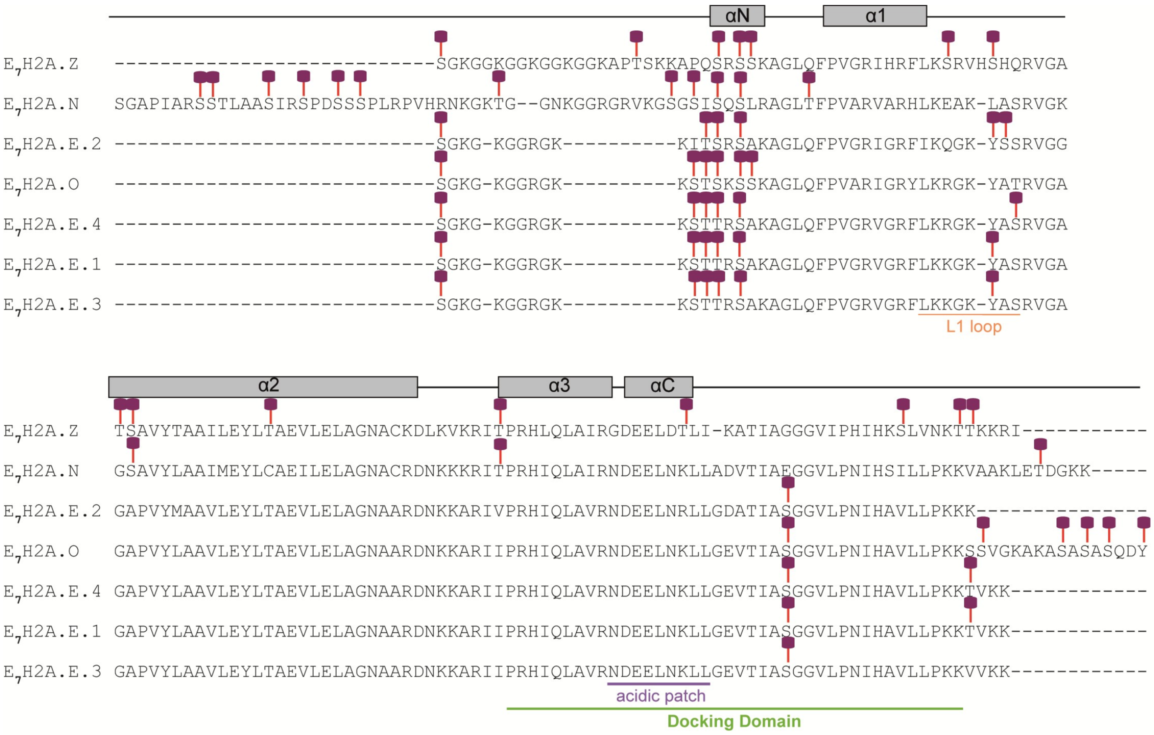

Fig. S8: Analysis of putative phosphorylations deposited on variants from *Ectocarpus* species 7*.*

Alignment of the *Ectocarpus* species 7 H2A variants. The L1 loop, acidic patch and docking domain are underlined in orange, purple and green, respectively. Purple squares represent predicted phosphorylation sites. E_7H2A.Es and E_7H2A.O variants have a phosphorylatable serine at position 102. The E_7H2A.N variant has several phosphorylatable serines in its long N-terminal tail.

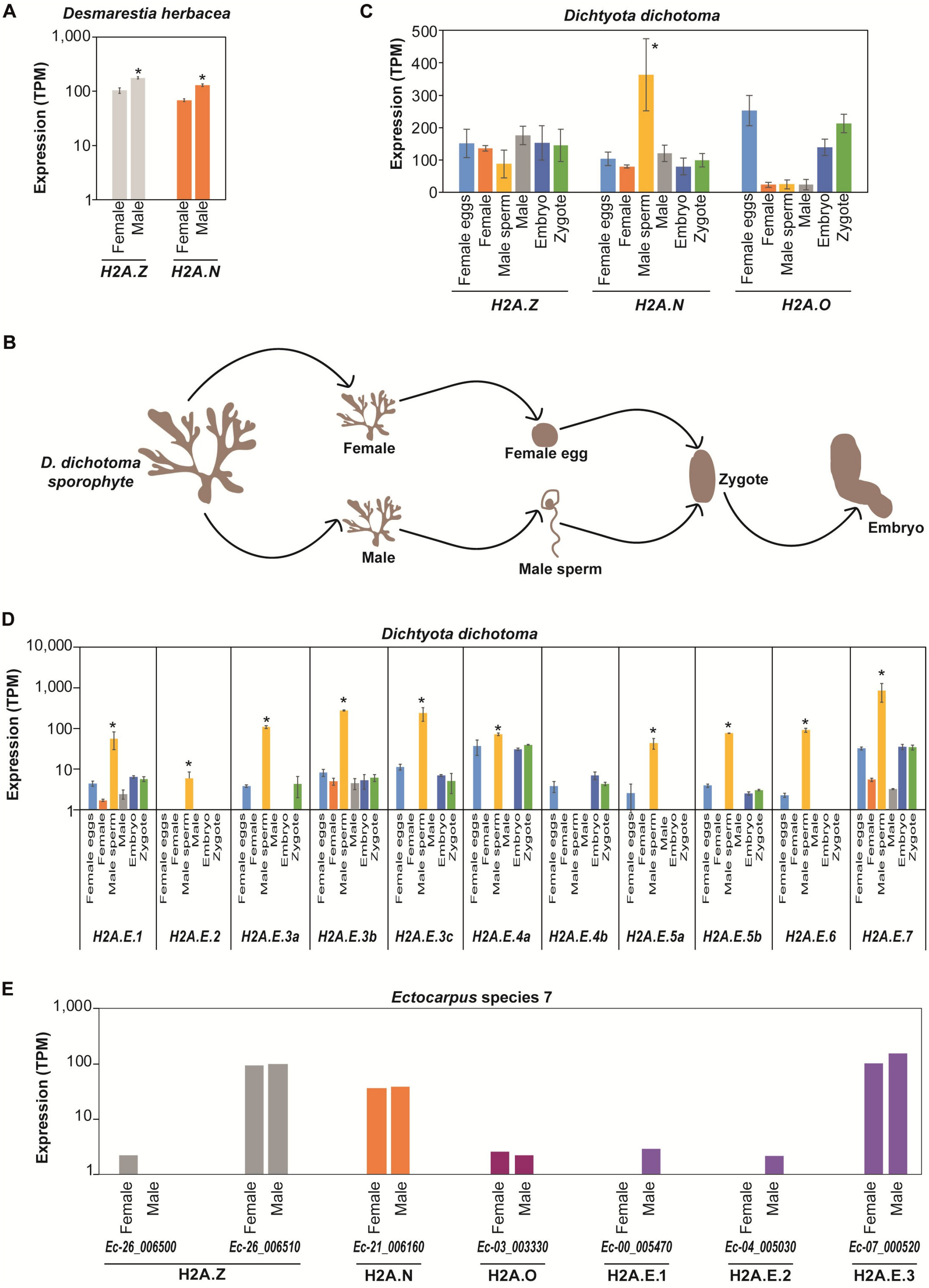

Fig. S9: Analysis of gene expression for the different H2A variants.

(A) Expression of *D. herbacea* H2A variant genes. The histogram represents the mean transcript abundance in TPM (Transcripts Per Kilobase Million) displayed with a logarithmic scale. It corresponds to RNA-Seq data obtained from three biological replicates consisting of independent

cultures of female and male gametophytes grown in low light and transferred one week before RNA extraction to high light to induce fertility. Only genes with a TPM value above 2 are displayed. *(B)* Schematic representation of the *D. dichotoma* life cycle. This species has a diplohaplontic life cycle with alternation of asexual thalli referred as sporophyte and sexual thalli referred as female and male gametophytes. Upon fertilization, sperm produced by the male gametophyte (referred as male) entered eggs produced by the female gametophyte (referred as female) producing zygotes 1h after fertilization. Embryos correspond to zygotes that undergo a first cell division 8 hours after fertilization and harbor a tip growth (Bogaert, Beeckman and De Clerck, 2017). *(C-D)* Expression of *D. dichotoma* H2A variant genes. The histogram represents mean transcript abundance in TPM (Transcripts Per Kilobase Million). It corresponds to RNA-Seq data obtained from three biological replicates consisting of independent cultures of female and male gametophytes (referred as male and female); eggs collected 15 min after release, sperm cells collected 1 h after release, zygotes collected 1 h after release and embryos collected 8 h after fertilization. Only genes with a TPM value above 2 are displayed. *(E)* Expression of the genes coding the *Ectocarpus* species 7 H2A variants. The histogram represents transcript abundance in TPM (Transcripts Per Kilobase Million). It corresponds to RNA-Seq data [63] obtained from male (Ec457) and female (Ec460) *Ectocarpus* species 7 strains. Gene IDs are displayed above the H2A variant they encode. Only genes with a TPM value above 2 are displayed. Student’s t test was performed to compare male sperm with the five other sample types; * $P < 0.05$.
