# Supplementary material for: Identification of novel H2A histone variants across diverse clades of algae
Source: Genome Biol. 2025 Sep 23;26:299. doi: 10.1186/s13059-025-03656-w (PMC12459048; doi:10.1186/s13059-025-03656-w)
Supplement: Supplementary file 3 — Additional file 3: Supplemental Methods. [file 13059_2025_3656_MOESM3_ESM.pdf]

## Supplemental Methods

### Sequence characteristics of RC H2A histones

To identify RC H2A histones encoded by the 22 chosen reference genomes from the Phaeoexplorer database, we looked for histone sequences that do not have the SQ[D/E]Y motif or H2A.Z features (*i.e.* a LEYLTAEVLELAGNA signature in the α2 helix, an L1 loop with a single amino acid insertion, an acidic patch with an extra acidic residue and a docking domain that is one amino acid shorter than the RC H2A sequence). Identified RC H2A histones were aligned to generate logos for the characteristic features. *H. akashiwo, C. australica* and *T. minus* RC H2As have a [K/R]SSKA motif in their αN helix (Additional file 1: Fig. S2A, Additional file 1: Fig. S3E center) and a similar motif was found in the αN helix of RC H2As from animals and plants (RSS[K/R]A, Additional file 1: Fig. S3E right). *H. akashiwo, C. australica* and *T. minus* RC H2As also have a conserved motif in their L1 loop: KxGRY[S/A]x (Additional file 1: Fig. S3F right). The RC H2As from *H. akashiwo, C. australica* and *T. minus* have a [L/M]EYLCAEILELAGNA signature in the α2 helix (Additional file 1: Fig. S3G right), an acidic patch with the signature DEELNKLL (Additional file 1: Fig. S2H right) and docking domains that end with a VLLPKK sequence (Additional file 1: Fig. S2A).

### Sequence characteristics of H2A.X variants

Identification of H2A.X was based on the presence of a SQ[D/E]Y motif at the end of the C-terminal tail and the absence of a H2A.Z signature in the α2 helix (Fig. 2B, Additional file 1: Fig. S2D). The H2A.X variants have a KQG[R/Q]Y[S/A][T/A/K] motif in their L1 loop (Additional file 1: Fig. S2E), a [L/M]EYLTAEVLELAGNA degenerated signature in their α2 helix (Additional file 1: Fig. S2F) and a DEELNKLL motif in the

acidic patch (Additional file 1: Fig. S2G). The H2A.X docking domain ends with a VLLPKK sequence and is 40 amino acids long (Additional file 1: Fig. S2D).

**Sequence characteristics of H2A.N variants**

H2A.N proteins were aligned to generate logos for characteristic features. The H2A.N variant has a long N-terminal tail with a PLRP motif (Additional file 1: Fig. S3B-D). The unusually long N-terminal tail shared no sequence homology with other proteins and included a disordered region according to Interproscan [52]. The αN helix of H2A.N has a QSLRA motif that is strongly divergent from the motifs of other H2A types such as RC H2As (Additional file 1: Fig. S3E). The H2A.N variant has a poorly conserved motif in the L1 loop (Kx[A/T]KLxx where x denotes any residue, Additional file 1: Fig. S3F left) and a LEYLCAEILELAGNA signature in the α2 helix (Additional file 1: Fig. S3G left). The H2A.N acidic patch signature is DEELNKLL (Additional file 1: Fig. S3H left) and it docking domain ends with a VLLPKK sequence (Additional file 1: Fig. S3C).

**Sequence characteristics of H2A.Z variants**

The H2A.Z proteins identified in the 22 selected reference genomes from the Phaeoexplorer database have a KSRVHSHQ motif in the L1 loop with a single residue insertion compared to the other H2A variants (Additional file 1: Fig. S4B), an acidic patch with an extra acidic residue (DEELDTLI, Additional file 1: Fig. S4C) and a highly conserved TTKKRI motif found in most C-terminal tails (Additional file 1: Fig. S4D). Their docking domains are one amino acid shorter than the RC H2A domain as expected for this variant (Additional file 1: Fig. S4D). We observed that Ectocarpales, Fucales, *S. latissima* and *D. herbacea* have identical H2A.Z proteins a (Additional file 1: Fig. S4D). Finally,

the H2A.Z protein of *U. pinnatifida* has a longer N-terminal tail and its branch length is therefore longer in the phylogenetic tree due to this sequence divergence (Additional file 1: Fig. S4A).

**Sequence characteristics of H2A.E variants**

The H2A.E proteins identified in the reference genomes from the Phaeoexplorer database have a KYAT-related motif in the L1 loop motif: KxGKY[A/S]x (Additional file 1: Fig. S5D). These H2A.E proteins have a DEELNKLL motif in their acidic patch (Additional file 1: Fig. S5E) and a docking domain of 40 amino acids ending with a VLLPKK sequence (Additional file 1: Fig. S5H). We defined four main phylogenetic clades for the H2A.E variant. The H2A.E proteins from these four classes differ by their αN helix (Additional file 1: Fig. S5F) and L1 loop consensus (Additional file 1: Fig. S5G): class 1 with RSAKA and K[Q/H]GKYAT motifs; class 2 with RSAKA and K[K/A/R]GKYAS motifs; class 3 with RSxKA and KxGKY[A/S]T motifs; class 4 with RS[S/A]KA and KxGKY[S/A][Q/S/K] motifs. Longer branch lengths were observed for some H2A.E variants in the phylogenetic tree indicating accelerated sequence divergence (Additional file 1: Fig. S5A-C). Such divergence was found in the all four classes (Additional file 1: Fig. S1B). The H2A.E variants from *D. mesarthrocarpum* and *C. tenellus* constitutes the class 1 of H2A.E variants (Additional file 1: Fig. S5A), their αN helix and L1 loop consensus of class1 being more conserved than those of the three other classes. Finally, the H2A.E variants from other brown seaweeds are dispersed in classes 2 to 4 (Additional file 1: Fig. S5A-C).

**Sequence characteristics of H2A.O variants**

The H2A.O proteins identified in the reference genomes from the Phaeoexplorer database have a KYAT-related motif in the L1 loop motif: KxGKYA[T/S] (Additional file 1: Fig. S6B). These H2A.O proteins have a DEELNKLL motif in their acidic patch (Additional file 1: Fig. S6C)

and a docking domain of 40 amino acids ending with a VLLPKK sequence (Additional file 1: Fig. S6D). The H2A.O variant is also characterized by a long stretch of lysine and glycine in its N-terminal tail, a KYAT-related motif in the L1 loop motif and a highly conserved SQDY signature in the C-terminal tail (Fig. 6A).

**Sequence characteristics of H2A variants in diatoms**

Features of RC H2A and H2A.X were similar between diatoms and brown seaweeds (Additional file 1: Fig. S7A). Brown seaweed and diatom H2A.Z proteins had conserved features but diatom proteins lacked the TTKKRI C-terminal motif present in brown seaweeds (in red, Additional file 1: Fig. S7B). Both of the chosen diatoms (Additional file 1: Fig. S7A-B) lack H2A.N, H2A.E and H2A.O variants.

**Sequence characteristics of H2A variants in the unicellular green alga *C. reinhardtii***

The *C. reinhardtii* predicted protein XP_001691545.1 is also referred as ch2a-IV / HTA2/HTA10 in [13] and H2A.0 in [15]. This latter study predicted five proteins with minor sequence variations (H2A.0, H2A.1, H2A.2, H2A.3, H2A.4, Additional file 1: Fig. S7F, divergent amino acids in red) and we thus speculated that they all correspond to XP_001691545.1. The XP_001691545.1 protein from *C. reinhardtii* has a KKGKYAE motif in its L1 loop and a H2A.Z signature in its α2 helix: we named it CrH2A.E (Fig. 7A). We used the name CrH2A to refer to A0A2K3DNT1 since it lacks any variant features (Additional file 1: Fig. S7C); it was referred as H2A.v2 in [15]. The protein CrH2A seemed to be incomplete based on its length (99 amino acids, Additional file 1: Fig. S7C). The proteins XP_001693700.1 and XP_001691141.1 were referred as H2A.v and H2A.v3, respectively in [15] and were named CrH2A.Z.1 and CrH2A.Z.2. indeed, they have a L1 loop with an insertion and a H2A.Z signature in their α2 helix, as well as an acidic patch with one supplemental acidic residue and a docking domain of 39 amino acids (Additional file 1: Fig. S7E). Therefore, the unicellular green alga *C. reinhardtii* has a RC H2A, two H2A.Z variants and a

H2A.E variant (Additional file 1: Fig. S7C & E), but lacks an H2A.X variant [15]. Note that both green algal *Ostreococcus* species (Additional file 1: Fig. S7C-E) lack H2A.N, H2A.E and H2A.O variants.

**Sequence characteristics of H2A variants in red algae**

The unicellular species *C. merolae* lacks an H2A.X variant but has the RC H2A and the H2A.Z variant. The XP_005537304.1 protein from the unicellular red alga *C. merolae* lacks any variant signature and was named CmH2A (Additional file 1: Fig. S7C). In this species, XP_005538662.1 has a H2A.Z signature in its α2 helix as well as one insertion in its L1 loop, an acidic patch with a supplemental acidic residue (DEEL**D**QLV) and a docking domain of 39 amino acids (Additional file 1: Fig. S7E): it was therefore named CmH2A.Z. The red multicellular alga *C. crispus* and the red unicellular alga *P. purpureum* both possess H2A proteins with a SQ[E/D]Y-like motif without a H2A.Z signature in their α2 helix. These proteins (XP_005715904.1 and XP_005710906.1 and A0A5J4Z1G0) were named CrrH2A.X.1, CrrH2A.X.2 and PpuH2A.X, respectively (Additional file 2: Table S1, Additional file 1: Fig. S7D). The red multicellular alga *C. crispus* and the red unicellular alga *P. purpureum* also had H2A proteins with a H2A.Z signature in their α2 helix: XP_005715231.1 and A0A5J4YL73 that we named CrrH2A.Z and PpuH2A.Z (Additional file 2: Table S1, Additional file 1: Fig. S7E). Both proteins have a L1 loop with an insertion typical of H2A.Z proteins, an acidic motif with a supplemental acidic residue and a docking domain of 39 residues (Additional file 1: Fig. S7E). The red unicellular alga *G. sulphuraria* has only one H2A protein that has H2A.E-like signatures. Indeed, it has a KNGNYAE motif in its L1 loop and a H2A.Z signature in its α2 helix (Fig. 7B).
